# Digital biomarkers for non-motor symptoms in Parkinson’s disease: the state of the art

**DOI:** 10.1038/s41746-024-01144-2

**Published:** 2024-07-11

**Authors:** Jules M. Janssen Daalen, Robin van den Bergh, Eva M. Prins, Mahshid Sadat Chenarani Moghadam, Rudie van den Heuvel, Jeroen Veen, Soania Mathur, Hannie Meijerink, Anat Mirelman, Sirwan K. L. Darweesh, Luc J. W. Evers, Bastiaan R. Bloem

**Affiliations:** 1 Radboud university medical center, Donders Institute for Brain, Cognition and Behaviour, Department of Neurology, Center of Expertise for Parkinson & Movement Disorders, Nijmegen, The Netherlands; 2 HAN University of Applied Sciences, School of Engineering and Automotive, Health Concept Lab, Arnhem, The Netherlands; 3 UnshakeableMD, Oshawa, ON Canada; 4 ParkinsonNL, Parkinson Patient Association, Bunnik, The Netherlands; 5 Tel Aviv University, Sagol School of Neuroscience, Department of Neurology, Faculty of Medicine, Laboratory for Early Markers of Neurodegeneration (LEMON), Center for the Study of Movement, Cognition, and Mobility (CMCM), Tel Aviv, Israel; 6 Radboud University, Institute for Computing and Information Sciences, Nijmegen, The Netherlands

**Keywords:** Parkinson's disease, Predictive markers, Neurodegenerative diseases

## Abstract

Digital biomarkers that remotely monitor symptoms have the potential to revolutionize outcome assessments in future disease-modifying trials in Parkinson’s disease (PD), by allowing objective and recurrent measurement of symptoms and signs collected in the participant’s own living environment. This biomarker field is developing rapidly for assessing the motor features of PD, but the non-motor domain lags behind. Here, we systematically review and assess digital biomarkers under development for measuring non-motor symptoms of PD. We also consider relevant developments outside the PD field. We focus on technological readiness level and evaluate whether the identified digital non-motor biomarkers have potential for measuring disease progression, covering the spectrum from prodromal to advanced disease stages. Furthermore, we provide perspectives for future deployment of these biomarkers in trials. We found that various wearables show high promise for measuring autonomic function, constipation and sleep characteristics, including REM sleep behavior disorder. Biomarkers for neuropsychiatric symptoms are less well-developed, but show increasing accuracy in non-PD populations. Most biomarkers have not been validated for specific use in PD, and their sensitivity to capture disease progression remains untested for prodromal PD where the need for digital progression biomarkers is greatest. External validation in real-world environments and large longitudinal cohorts remains necessary for integrating non-motor biomarkers into research, and ultimately also into daily clinical practice.

## Introduction

Since the last decade, the quest for disease-modifying therapies in Parkinson’s disease (PD) has intensified^1^. Measuring clinical disease progression accurately and objectively during these trials is of great importance to assess treatment efficacy. Currently, gold-standard outcome measures for such trials are the Movement Disorders Society scales for motor symptoms and daily functioning^2^. Use of these scales comes with several challenges: they require substantial assessment time, are conducted episodically and are based on subjective interpretation, which leads to considerable measurement errors over time^3^.

In the past decade, we have seen tangible advancements in the remote digital assessment of the motor symptoms of PD, both in clinical practice and in research settings^4–8^. Digital sensors can monitor symptoms non-invasively and often passively (without need for active human intervention). The quantification of symptoms and signs at a higher frequency and for longer time frames (even continuously for several digital outcomes) makes such digital biomarkers a more objective, more convenient and potentially more sensitive alternative to the episodic in-clinic assessment of disease progression. As such, several digital biomarkers of motor signs have been included in research settings^9–11^.

Much less attention has been paid to non-motor symptoms, in both research and clinical practice, apart from olfactory dysfunction as a supportive criterion in the MDS diagnostic criteria^12^. Yet, non-motor symptoms have a substantial negative impact on quality of life in affected individuals^13^, precede motor symptoms during the prodromal phase up to two decades^14^ and are subject to daily fluctuations just as motor symptoms, favoring longitudinal measurement over episodic in-clinic assessment^15^. The earlier occurrence of non-motor symptoms during the prodromal phase makes them potentially more sensitive as progression biomarkers in disease-modifying trials in prodromal populations and more suitable as diagnostic biomarker for the inclusion of at-risk or prodromal individuals in trials, and also as diagnostic marker in clinical practice. Having access to digital biomarkers that can objectively and remotely monitor non-motor symptoms across the spectrum of disease severity paves the way for more patient-relevant and unobtrusive outcomes in research. The importance of such personalization is further emphasized by the recent Food and Drug Administration (FDA) guidelines on patient-focused digital health and drug testing^16,17^.

In this review, we provide an overview of the current developmental status, validity and reliability of digital, passive non-motor biomarkers for the prodromal and clinically manifest phase of PD. In contrast to previous reviews that were limited to PD studies^18–20^, we did not restrict our search strategy to the PD field, as we recognize that various non-motor symptoms may also be present in other diseases. Therefore, we aimed to investigate whether digital biomarkers that have been developed in these other areas might also have relevance for the PD field. In addition, we will focus on both diagnostic and progression non-motor biomarkers and critically appraise the feasibility of implementing specific non-motor biomarkers in trials. Lastly, we make recommendations for future research based on our review.

## Results

### Selection of sources of evidence

**Fig. 1:**
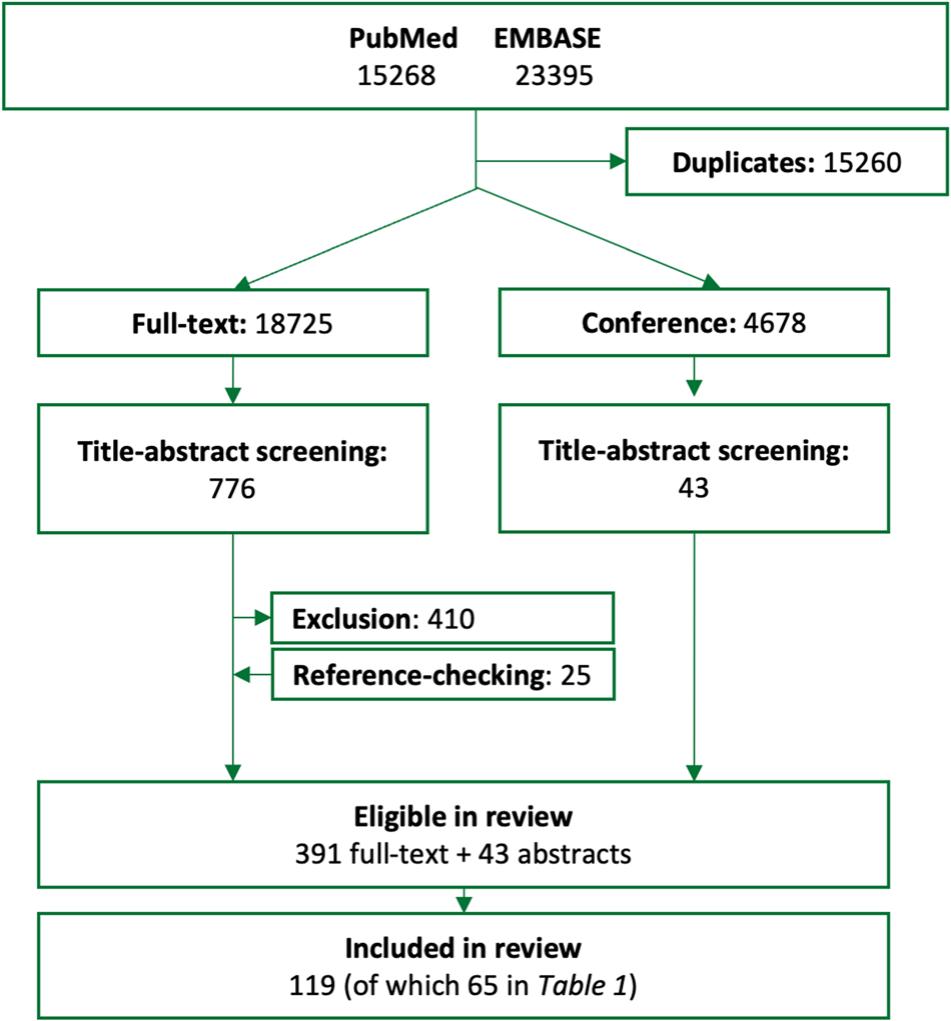
Flowchart of the article selection process.

From PubMed and EMBASE, a total of 18,725 articles and 4678 conference abstracts were screened. 391 articles were eligible, and 119 were eventually included after full-text screening. The flowchart in Fig. 1 depicts the article selection process. The most important reasons for exclusions after title-abstract screening were that a biomarker did not measure the intended symptom or that the measurement of a biomarker was not (fully) passive. A reference manager (Endnote®) library file outlining the complete search strategy and final selection is available from the authors.

### Characteristics of sources of evidence

Of the 20 PD non-motor symptoms, seventeen had one or more proposed passive digital biomarkers. Eight of the 65 studies included in the main table (Table 1) included individuals with PD, and two included individuals with RBD. No other prodromal PD populations were included. Of all eligible studies, most focused on measures of sleep (*n* = 97), heart rate variability (*n* = 79), mood (*n* = 71) and cognition (*n* = 53). For hallucinations, one study was included. No studies reported eligible digital biomarkers for olfactory dysfunction, erectile dysfunction or color vision disturbances.

**Table 1:** Digital biomarkers of non-motor symptoms currently available or under development

Prodromal symptom	Measured modality	Device	Study design and population	Validity	Reliability	Feasibility (caveats, ease-of-use, cost)	Literature on longitudinal change^a^	Refs.
*Sleep and fatigue*								
Sleep disturbance	(Im)mobility during sleep	Accelerometer (Park’nson’s KinetiGraph®)	Observational study. *N* = 273 (155 control, 72 PD, 46 who underwent PSG of which 36 with sleep disorder). 6 consecutive days of recording. First night in-clinic with PSG, subsequently at home.	Specificity 86%, sensitivity 80% of detecting normal vs. abnormal PSG. Sleep score of KinetiGraph® associates with disease severity (*p* = 0.025, unclear effect size). Sleep efficiency, fragmentation, and quality are most important predictive factors.	N/A	Commercialized product, but not yet as feature-specific sleep tracker. Wearable as watch. Unobtrusive, high compliance indicating ease of use.	N/A	^22^
	Circadian rhythm monitoring	Actigraphy (Kronowise 3.0)	Early validation study against PSG, *N* = 30 (including 15 PD). 7 consecutive days of recording at home (24 h/day) vs. 1-night in-clinic PSG.	Smartwatch shows excellent correlation with PSG on time in bed (*r* = 0.94), total sleep time (*r* = 0.76), sleep efficiency (*r* = 0.70) and time awake after sleep onset (*r* = 0.62). PD participants showed increased sleep fragmentation and movement.	N/A	Commercialized product that measures multiple features. Unobtrusive, easy to use wrist watch.	N/A	^23^
	In-bed movement, sleep fragmentation	Tri-axial accelerometer on lower-back (Axivity AX3® or DynaPort MiniMod Module®)	Observational study, *N* = 510 (305 PD, 205 healthy control). ≥2 days at-home measurement.	More upright periods and lower turn velocity are associated with increased disease severity (*p* ≤ 0.021). Turning time and magnitude distinguishes controls from early PD (*p* ≤ 0.002). Reduced nocturnal movement associates with increased motor severity.	N/A	Tested in early PD (<1 yr after diagnosis). Commercialized product worn on lower back. Cumbersome as manual dexterity is required to attach. Does not account for nocturnal akinesia.	N/A	^24^
REM-sleep behavior disorder	Movement distribution (*‘RBDAct’* algorithm)	Wrist actigraphy (GENEActiv Original®)	Early validation study, *N* = 26 PD, *n* = 18 controls with insomnia. 2-week at-home measurement of sleep-wake patterns.	Differences in sleep stage N3 and apnea-hypopnea index between RBD and non-RBD in PD. Accuracy of 92.9 ± 8.16% in-clinic to diagnose RBD in PD, 100% over a 2-week at-home period. 6.8% misclassification when including controls with insomnia.	N/A	Unobtrusive, easy-to-use wrist worn sensor. Affordable price. Automated data processing with results that are easy to interpret.	N/A	^33^
	Rest-activity cycle using I < O index (% motor activity in bed vs. out of bed)	Wrist actigraphy (Micro Motionlogger Watch)	Late validation study, *N* = 94 (incl. 19 iRBD, 16 healthy control, rest obstructive sleep apnea and restless legs syndrome). 2-week watch sleep measurement, video-PSG as gold-standard.	I < O index can classify RBD from other groups with 82.4% accuracy, sensitivity of 63.2% and specificity of 89.1%.	Significant performance change with different sensor positions.	Commercialized, unobtrusive product. Multiple features measured. Easy to use wrist worn sensor. Event marker available.	N/A	^29^
	Immobile bouts	Wrist actigraphy (MotionWatch®)	Early validation, *N* = 35 (14 RBD, 21 other sleep disorders). One night in-clinic versus PSG.	Sensitivity of 61%, specificity of 89% to detect RBD.	N/A	Easy-to-use wrist-worn sensor.	N/A	^30,36^
Excessive daytime sleepiness	Immobile bouts (nap detection)	Actigraphy (SleepWatch-O®)	Longitudinal study, *N* = 2920 elderly. At-home measurement of nap duration for at least five consecutive days.	Nap duration >1 h associated with disease risk after 11 years (OR 1.96 (1.25–3.08)). This associations was stronger when people also reported excessive daytime sleepiness based on the gold-standard Epworth Sleepiness Scale (OR 2.52, (1.21–5.27)).	Correlation is independent from circadian instability.	Commercialized and available. Easy to use wrist worn sensor.	Prodromal excessive daytime sleepiness (>2 years) correlates with manifest PD, 11 years longitudinal follow-up.	^40^
	Immobile bouts (nap detection)	Triaxial accelerometer (Parkinson’s KinetiGraph®)	Observational study, *N* = 68 PD. Ten-day measurement period (*n* = 68 PD) vs. one-day PSG (only available for subset of seven people with PD).	85% concordance of immobile bout detection between sensor and PSG (classification of bouts: sensitivity of 83%, specificity 89%). Good correlation with excessive sleepiness subclasses (*p* = 0.01).	Independent from bradykinesia, but dependent of levodopa which increased naps in 53% of individuals.	Commercialized product, but not yet as sleep tracker. High ease of use. Wearable as watch. Additional medication reminder.	N/A	^38^
	LF/HF ratio	Wrist photoplethysmography (Empatica E4)	In-lab validation of photoplethysmography sensor against subjective drowsiness levels, *N* = 30 healthy controls.	Validated for driver drowsiness: 92% accuracy to detect drowsiness; comparable accuracy as medical-grade electrocardiogram.	Not tested in mobile or uncontrolled circumstances.	Commercialized available wrist device.	N/A	^39^
Fatigue	HRV (mean normal-to-normal intervals, total spectral power and low frequency)	Wearable single-channel ECG (LaPatch®, sensor ADS1292R)	*N* = 35 controls. In-lab one-day measurement. Reference is Chalder Fatigue Scale as manipulated by answering quiz questions.	Accuracy to detect fatigue state is 75.5%, AUC 0.74.	Not tested in mobile or uncontrolled situations.	Sensor connected via Bluetooth to phone. More complicated use, attached via electrodes on skin. May translate to other commercially, widely available wearable electrocardiogram sensors in existing devices.	N/A	^42^
	HRV, respiration rate and skin impedance	Self-developed single-channel electrocardiogram (AD8232, *SparkFun*), strain sensor on chest and skin impedance sensor on hand-palm	Proof of concept, *N* = 3 healthy controls. In-lab 24-h measurements with sensors vs. compound score of self-report and stress test performance.	Accuracy to detect fatigue of 84–89% depending on machine learning model.	N/A	Two separate modules, intrusive sensor on hand-palm.	N/A	^43^
*Autonomic dysfunction*								
Pain	Skin impedance	Wrist galvanic skin response (Empatica E4)	Early validation, *N* = 20 postoperative patients. NRS (1–5) for pain score after induced personalized low-intensity painful triggers (walking, coughing). 30-min device measurements.	Accuracy to detect pain decreases with increasing pain score (0 to 1 = 86%; 1 to 2 70%, 2 to 3 = 61.5%, etc.).	Tested in controlled conditions. Higher pain levels associate with higher motion artifacts.	Easy to use watch. Unclear whether pathological skin impedance affects the performance of this method.	N/A	^46^
	Facial micro-expressions and voice, movement, behavior, activity and body signals	Mobile application (ePat®)	Early validation, *N* = 40 with dementia. Application measures compared against Abbey Pain Scale (rated by caregiver) during various moments at rest or post-movement.	High concurrent validity with Abbey Pain Scale if combined with questionnaires in mobile applications (*r* = 0.88). Early signs of appropriate discriminant validity (no statistics).	Inter-rater reliability of 0.74 (Cohen’s κ).	Potential difficulty to extrapolate to PD due to hypomimia. Potentially difficult software to operate. Not passive collection of images (yet).	N/A	^47^
	Daytime motor patterns, specific sleep parameters during nighttime, frequency and intensity of movement	Accelerometer on right hip (*Actical Step*, Mini-Mitter)	Early validation, *N* = 68 HIV patients. 1-week measurements with sensor validated against self-reported current and chronic pain (chronic using Brief Pain Inventory).	Correlation between sensor and self-report was 0.69 for pain severity. Cohen’s κ (agreement) for chronic pain detection by sensor vs. Brief Pain Inventory was 0.49. Accuracy 75%, sensitivity 70%, specificity 78%.	N/A	Difficult to extrapolate to PD due to motor symptoms (neurological comorbidity was exclusion criterion). PD-related insomnia might interfere.	N/A	^48^
Reduced heart rate variability	Heart rate variability (time frequency, high-frequency and LF/HF ratio)	Ambulatory electrocardiogram monitor (Fukada-Denshi)	Observational study, *N* = 47 (17 PD, 30 healthy controls) with 24 h monitoring of heart rate.	HRV spectra (HF, LF/HF, TF) differ between control, early and advanced PD (i.e. sensitive to disease severity).		Ambulatory electrocardiogram device not technically specified.	N/A	^56^
	Heart rate variability (variety of frequency and time domain estimates)	Multiple sensors: Ring (OURA) Chest patch (Cortrium C3) Wristwatch (Empatica E4) Chest straps (Firstbeat, Zephyr BioHarness, Polar H10 with HRV4Training or ELT-ECG app)	Late validation against gold standard or mobile ECG in both laboratory and at home situation, range *N* = 5–74 healthy controls. Situations range from resting state, paced breathing to activity (cycling).	Oura Ring has low mean standard absolute percent error from gold standard during rest for RMSSD (6.84%; concordance correlation coefficient: 0.91). When using chest-based straps, Polar H10 with HRV4Training or ELT-ECG apps had higher error percentages (4.1–7.7%), but still lower than Firstbeat (11.3%)^52^ Cortrium has fair ICC in active conditions (0.53–0.70). In free-living conditions, CV increases to 35%^53^. At rest, Empatica E4 has low predictive estimates for rMSDD, and fair estimates for SDNN, LF and HF^55^. In a different study, Empatica E4 (average 0.27) and Zephr BioHarness (average 0.42) have low R2 correlation values with gold standard in active (cycling) conditions^54^.	Test-retest reliability across 14 days for Cortrium was 0.53–0.90 (ICC). OURA has high agreement with gold-standard (r^2^ −0.98–0.99)^62^. Polar shows good ICC against the gold standard (ICC ≥ 0.8^49^, >0.75^51^ and *r* > 0.97^50^), with some negative influence of bodily orientation^50^. Cortrium shows compliance of 73% over 14 days of at-home measurement.	Oura Ring is easy to wear, attractive reasonably compact shape. High risk of movement artifacts. Cortrium is an easy to wear chest patch, commercially available and user-friendly. Empatica is highly feasible, wristband that is easy to wear, transmits data wirelessly and requires no further equipment.	N/A	^49^ ^50^ ^51^ ^52^ ^53^ ^54^ ^55^ ^62^
	Heart rate variability (variety of frequency and time domain estimates)	Chest straps (Polar RS800G3 and Polar S810i)	Early validation, *N* = 30 healthy controls, In-lab measurement of sensor compared against gold standard ECG.	Chest straps show strong correlation with gold standard (>0.86) across different measures of heart rate variability: standard deviation of NN intervals, RMSSD, LF, HF and LF/HF.	N/A	Chest straps are commercially available. Easy to equip but can be inconvenient to wear for longer periods.	N/A	^57^
Bladder dysfunction	Urine flow rate	Smartphone microphone	Early validation against standard uroflowmetry, *N* = 44 healthy males, in clinic standard uroflowmetry with synchronous sound recording.	Very high correlation for flow rate with golden standard =0.96–0.99, although curve of correlation is not similar, possibly due to sound artifacts. An	N/A	Scalable and cheap solution, but only tested for males in standardized situation.	N/A	^68^
	Urine analysis, urine flow rate, and stool classification	Urinalysis through test strips; motion sensor, video camera for urine stream flow and stool analysis; finger- and analprint for biometric user identification (‘smart toilet’)	Proof-of-concept testing of the video-based uroflowmetry against gold standard (*N* = 10) and testing accuracy of stool classification system (*N* = 11)	Accuracy of uroflowmeter comparable to gold standard. Results of machine learning algorithms for stool classification comparable to surgeons’ judgement in ~75% of cases.	Synthetic urine used as calibration solution at start of measurement period.	Extensive sensor suite integrated in toilet, focused on passive, non-invasive, and affordable monitoring. Fits currently existing toilets.	N/A	^66^
	Bladder filling	Lower abdomen near-infrared spectroscopy	Proof-of-concept, measurement of six urination events in-clinic on one healthy individual.	Pre- and post-voiding succesfully detected (mean of the between-period differences: 0.022 with standard error of mean of 0.0096).	N/A	Wearable device with wireless data transmission. Large device and attachment with tape are potentially uncomfortable. Not helpful for urge incontinence.	N/A	^67^
Erectile dysfunction	Nocturnal penile tumescence and rigidity	Tip and base penile loops that slightly tighten and release (RigiScan®)	Validation^188^ against gold standard, *N* = 95 (77 with erectile dysfunction). Sensor distinguishes different erectile dysfunction types. Two- or three-night measurements. Second study (*N* = 172) investigates responsiveness to sildenafil effects during three nights^165^	Accurately detects specific type of erectile dysfunction in 83.7% of cases^188^. Sensor is sensitive to sildenafil effects (accuracy 77.3%, AUC = 0.86)^165^	N/A	Best option available to measure erectile dysfunction, but intrusive, somewhat older sensor.	N/A	^165,188^
Low skin impedance	Skin impedance	Smartwatch (Empatica E4®)	Early validation of smartwatch against gold standard and benchmarking of wearable during in-lab cycling, *N* = 18 healthy participants.	Comparison against gold standard showed reasonable but not high levels of correspondence (no formal statistical tests available). Sensor is also used as proxy for pain in different study^46^.	N/A	Easy to wear. Wristband transmits data wirelessly and requires no other equipment. Skin impedance accuracy highly influenced by adequate placement including skin contact.	N/A	^54^
	Skin impedance	Custom-build ring system measuring electric resistance of the skin	Proof of concept with validation against gold standard (*N* = 4 healthy adults) during 3 stress scenarios lasting five minutes each.	Reasonable comparison with gold standard, except for amplitude of skin impedance (>43% mean percentage variation)	N/A	Possibility of integration in larger wearable system, but ring is large, potentially uncomfortable. Early conceptual phase.	N/A	^58^
	Skin impedance	Shirt-implemented sensor as part of multimodality vest (‘Smart Vest’)	Proof of concept in lab, no validation. *N* = 25 healthy men, 30-min vest wearing during standing and walking.	Reasonable accuracy, data does not yet support medical application due to low-quality data.	N/A	Concept is feasible, opportunity for multi-sensor configuration. Early conceptual phase.	N/A	^60^
Hyperhidrosis	Sweat rate	Wearable microfluidic patch and smartphone image processing to analyze colorimetric patch	Early validation and reliability testing, *N* = 312 athletes, by comparing patch based sweat rate against gold standard (absorbent patch) during in-lab and on-field exercising.	Accuracy during exercise: r = 0.83 between both patches (*n* = 43). Sweat rate correlated well with gold stanadard (*r* = 0.90). Prediction of whole-body sweating rate (*n* = 312): R^2^ = 0.74.	Two-day measurement for *n* = 12, comparable measurement variation between absorbent (CV = 8.8% sweating rate, 12.8% sweat) and microfluidic patch (CV = 8.9% sweating rate, 11.6% sweat)	Sports-focused, patch fell off sometimes or not enough sweat extracted for evaluation. Makes generalization to at-rest situation in PD difficult. Inconvenient and non-passive interaction needed to take photograph of patch.	N/A	^70^
	Sweat rate	Wearable microfluidic patch with electrochemical and electrical sweat sensing electrodes	Proof of concept, *N* = 5 healthy controls. Sweat measurements during in-clinic and real-world scenarios, e.g. working or after eating dopamine rich foods.	Convergent validity: sweat rate correlates with heart rate across 24 h (across two trials). No statistics reported, proof-of-concept study.	N/A	Sweat rate measurements are location-independent providing convenience. Patch is small and flexible, but placement must be accurate. Real-time sensor but lacks automatic data transfer.	N/A	^71^
	Sweat rate	Ear-worn impedance sensor	Proof of concept, *N* = 4 healthy controls. In-lab calibration of device and controlled testing with 20-min indoors cycling exercise.	Accuracy/calibration: Results as expected in controlled lab experiment. Face validity: Testing on cycle demonstrates expected results, e.g., more sweat when exercising. No statistics reported, proof-of-concept study.	N/A	Sensor is still in early development stages, with some design-related issues incl. sensor displacement when moving and impedance level not resetting completely after exercise.	N/A	^72^
Aberrant skin temperature rhythm	Skin temperature	Smartwatch (Kronowise 3.0)	Proof of concept, *N* = 24 (12 PD), measurement duration is one week.	Sensitive to skin temperature changes throughout the day (not statistically analyzed)	N/A	Commercialized product, already deployed in trials. Easy to use.	N/A	^75^
	Skin temperature	Patch with thermometer on upper chest	Early validation, *N* = 35 healthy adults. Patch compared to gold-standard infrared forehead measurements, 24-h measurement duration.	Patch measures lower temperature (0.6 ˚C) than forehead sensor. When predicting state above/below 37.5 C, sensitivity = 0.50, specificity = 0.89.	Correlation with forehead measurements is weak and fluctuates across the day. Potentially due to self-administration errors of forehead measurement.	Wireless, small, flexible, unobtrusive patch that rarely irritates. Promising, but early conceptual phase. Highly dependent on accurate placement.	N/A	^76^
	Skin temperature	Semiconductor temperature sensor with built-in memory (iButtons®)	Proof-of-concept study with validation against gold standard in cold and hot environment (*N* = 6), and related skin temperature to objective sleep measures with smartwatch (*N* = 2).	Sensor slightly underestimates temperature (0.6 C) in colder environments but high mean accuracy (−0.09 degrees Celsius, up to −0.4) without calibration. High precision 0.05 degrees Celsius (up to 0.09)	Calibration further increases accuracy and precision.	Commercially available wireless, sturdy but relatively large sensor that is easy to clean and can measure for up to 34 h. Low sampling rate no issue for long-term monitoring. Uptake in circadian rhythm papers^189^	N/A	^77^
Orthostatic hypotension	Cuffless blood pressure measurement, photoplethysmography	Wrist-worn sensor (Biobeat® BB-613WP)	Validation study, *N* = 1057 with direct comparison against cuff-based gold-standard. Two additional measurements during exercise in *n* = 491.	Mean difference –0.1 ± 3.6 mmHg for systolic and 0.0 ± 3.5 mmHg for diastolic blood pressure. High interclass correlation coefficient ≥0.96 for both systolic and diastolic pressure. Low bias introduced by hypertensive or hypotensive patients (up to –0.63 mmHg).	N/A	Requires baseline calibration with cuff-based device necessary at the start of measurement period.		^80^
	Cuffless blood pressure using pulse wave analysis	Wrist-worn bracelet (Aktiia® 24/7)	Validation study, *N* = 91 individuals with wide range of mean blood pressures. Total 731 measurements against the reference gold standard cuff-based measurement in a protocol while lying, sitting, standing and exercising, once per week for 4 weeks.	Means + SD were 0.46 ± 7.75 mmHg different in sitting position, –2.44 ± 10.15 mmHg in lying position and –0.62 ± 12.51 mmHg in standing position for systolic BP. Diastolic values were slightly better in sitting and lying, but worse in standing position.	In standing position only 45% of blood pressures reliably measured, up to 78% when in lying position (low percentage of valid measurements).	Baseline calibration with cuff-based device necessary at the start of measurement period. Participants were satisfied with novel device compared to traditional methods.	N/A	^81^
	Arterial blood pressure using tactile sensor with pneumatic bladder	Finger-wearable device	Proof of concept, *N* = 97 controls. Training (*n* = 49) and validation (*n* = 19) data for blood pressure algorithm development and test data (*n* = 29) to determine the estimation accuracy compared to brachial dual-observer auscultation.	255 measurements. The finger-wearable device generates estimates comparable to the gold standard, with a mean error of 0.9 mmHg (SD 6.9) systolic and −3.2 mmHg (SD 7.0) diastolic.	N/A	Fit: 20% had device on a different finger, for 57% the device was too big. Potentially uncomfortable to wear in daily life.	N/A	^79^
*Gastro-intestinal disorders*								
Constipation	Colonic transit time	Wireless motility capsule (Smartpill®)	Observational study, *N* = 66, PD with chronic constipation (fulfilling Rome criteria for constipation).	62% of cohort had colonic transit delay. Transit times correlated with constipation scores (*p* = 0.01), but no correlation with PD severity or duration.	N/A	8% was unable to swallow the pill, 6% had technical issues with data transfer.	N/A	^86^
	Colonic motility and transit times (velocity, length of propagation)	Electromagnetic ingestible capsules (Motilis 3D-Transit®)	Proof of concept, 34 recordings in healthy adults, 7 patients with chronic (carcinoid) diarrhea.	Significant difference in transit times with chronic diarrhea (*p* = 0.04), Allows for detailed pattern assessment of colonic motility. No validation.	High percentage of non-usable recordings (24%) due to recording issues or poor recording quality.	Potentially uncomfortable to wear (receiver worn on lower abdomen). Potential interference from electronic devices.	N/A	^84^
	Myoelectric intestinal activity	Wireless Patch System (WPS, G-Tech Medical) attached to lower abdomen, connected to smart device (Apple iOS)	Validation, *N* = 108 PD, for three days once yearly, up to three years, compared to a synchronous reading by wireless motility capsule (Smartpill®).	High level of concordance with intra-luminal transit measurement by motility capsule (no statistical tests). Shows sensitivity to meals and metoclopramide	High reproducibility of colonic transit pattern of specific individuals over three-year duration.	Non-invasive, not interfering in daily life. Requires phone interaction.	N/A	^85^
Dysphagia and esophageal dysmotility	Submental sound and movement	Combination of submental mechano-acoustic patch and mechano-acoustic chest sensor	Proof of concept, *N* = 19 (10 dysphagia, 9 controls). 321 swallows from patients, 184 from controls. In-clinic assessment. Single recording for controls, 2 recordings per patient, one within 1 week of admission, one within 1 week before discharge.	Sensitivity and specificity ~80% / 60%, in classifying dysphagia. Respiratory-swallow coordination most important feature.	N/A	Flexible, wireless sensor on throat. Visible and disturbing to wear. Continuous phone presence necessary.	N/A	^90^
	Electrical activity in swallowing muscles	Surface-electromyography (sEMG)	Early validation study, *N* = 40 healthy adults. Comparison between sEMG tailored to head-neck region and conventional sEMG.	809 swallows analyzed. The mean signal-to-noise ratio of the sEMG signal acquired with the experimental patch (Mleft = 20.64 and Mright = 20.31) was higher than the mean SNR of the signal acquired with the conventional electrodes (Mleft = 19.44 and Mright = 19.65)	N/A	Limited wearability, electrodes on throat uncomfortable and visible. Satisfaction and comfort level were comparable between experimental patch and conventional electrodes.	N/A	^92^
	Swallowing sound	Smartphone-based device with microphone worn around the neck (Swallowscope®)	Proof of concept study comparing 70 people at-risk of dysphagia with 15 healthy controls. Controls performed dry/saliva swallows, people with presumed dysphagia underwent videofluoroscopic examinations.	71 swallows, 69 recognized by study device with 2 false negative and 14 false positive. Precision of 83.7% (range: 66.6%–100%) and a sensitivity of 93.9% (range: 72.7%–100%)	N/A	Limited wearability, continuously visible around the neck. Easy to wear.	N/A	^91^
*Neuro-psychiatric features*								
(Global) cognitive deficits	Amplitude of 24-h activity rhythm, peak activity time, interdaily stability of activity rhythm, intradaily variability of activity per hour.	Wrist-worn actigraphy (Actical®)	Longitudinal cohort, *N* = 1401 elderly (mean age 81 years), at home. Annual 10-day wearing period. Follow-up maximum 15 years.	Lower activity rhythm amplitude (HR 1.39 and 2.09), high intradaily variability (HR 1.22 and 1.97) is associated with AD conversion in healthy and MCI, respectively. Decreased interdaily stability is associated with conversion of MCI to AD. Similar articles: - Actigraphic rest-activity rhythm associates with early-onset dementia^94,96,109^. - Rest-activity rhythm amplitude and robustness are associated with later-onset dementia and MCI^95^.	Associations are not determined by sleep problems or comorbidities.	Concerns dementia in AD, difficulty extrapolating to PD.	Amplitude (*r* = 0.57) and interdaily stability (*r* = 0.31) positively correlate with cognitive changes longitudinally, intradaily variability correlates negatively (*r* = −0.52). Rest-activity rhythm was associated with cognitive decline 1 year^94^, 3 years^96^ and 5 years^95^ later.	^93^ ^94^ ^95^ ^96^ ^109^
	Walking speed and walking speed variability	Motion sensors placed in multiple rooms (*MS16A X-10*)	Early validation, *N* = 93 (54 healthy elderly, 31 nonamnestic MCI, or naMCI, 8 amnestic MCI, or aMCI). Mean follow-up 2.6 years. MCI assessed by Petersen criteria.	aMCI and naMCI differ in baseline walking speed. naMCI slower in-home walking, and higher decline in walking speed over time. Similar algorithm using cyclomatic complexity has AUC of 77% and predicts MCI onset 6 months before diagnosis^99^.	High inter-individual variability (individual deterioration most important). Limitation in PD because of motor features influencing walking speed.	Completely passive (no devices interaction or wearing), attached to ceiling. Only possible in single-person households without pets.	Slow walking speed (odds 8.76) and higher walking speed variability are sensitive markers to progression to naMCI.	^97^ ^99^
	Nap detection	Wrist actigraphy (*SleepWatch-O*)	Observational. *N* = 2751, older men, 5-day sensor measurement, longitudinal cognitive evaluation for 12 years using Trails B (processing speed test) and Modified Mini-Mental State examination (3MS).	Long nap duration ( > 2 h) is associated with dementia phenoconversion (OR 1.66 95%CI 1.09–2.54) and correlates with cognitive test performance (*p* ≤ 0.02, no correlation reported).	Sleep quality did not modify the established association.	Non-intrusive sensor. Necessity to wear all day. Battery performance unclear.	Nap duration predictive of cognitive worsening 12 years before phenoconversion	^98^
Anxiety	Accelerometry, blood-volume pulse, skin impedance, skin temperature and heart rate to measure acute psychological stress during physical activity.	Wrist-worn sensor (Empatica E4®)	Early validation study, *N* = 34 (healthy controls), in several different contexts ranging from rest to exercise. Anxiety measured by State-Trait Anxiety Inventory	Average accuracy of acute psychological stress detection during sedentary state 97.3%, during treadmill running 94.1% and stationary bike 84.5%. 87.2% accuracy in detecting acute psychological stress across all experiments.	More data needed to train the algorithm and testing the performance of deep learning approaches. Accurate representation of daily life by including different activity levels.	Easy to use and wearable long-term, also during physical activity. Multi-sensor integration.	N/A	^110^
	Wake-sleep rhythm	Wrist-worn actigraphy (Mini Mitter Actiwatch©-64), and smartphone	Longitudinal study, *N* = 265 anxiety and panic disorder. 9–14 years after baseline anxiety assessment, they performed one-week actigraphy which was used to predict anxiety after a total of up to 18 years follow-up.	Out-of-sample cross-validation demonstrates that the algorithm predicts symptom deterioration in individuals across a 17–18 year period with 84.6% sensitivity, 52.7% specificity, area under the curve = 0.696, accuracy = 68.7%.	Wake-sleep rhythm data collected across seven consecutive days. Prediction of symptom deterioration significant across 17–18 years.	Highly wearable, battery capacity unclear, uses proprietary software.	Actigraphy predicts anxiety longitudinally (up to 17–18 years).	^111^
	Linguistic property of voice-recorded words	Smartphone microphone	Proof-of-concept study, *N* = 86 healthy. Smartphone-based recognition of English words in natural speech are recorded during two weeks. Words are categorized and analyzed by linguistic software.	Higher levels of anxiety were associated with reward-related words (*r* = −0.29, *p* = 0.007) and vision-related words (for social anxiety, *r* = 0.31, *p* = 0.003).	N/A	Words analysis from voice recording potentially intrusive. Audio recordings of 15 seconds every 5 min, potential missings. No gold-standard reference of anxiety.	N/A	^112^
Depressive symptoms	Smartphone use entropy, regularity and variance	Smartphone (Android) use data gathered by a mobile sensing app (Carat®)	Longitudinal study, *N* = 629 non-specific cohort of adults. Passive smartphone data collection for 14 days. Behavioral markers analyzed using classifier algorithms. Depressive symptoms rated on Patient Health Questionnaire-8.	Accuracy of classifiers up to 95–98%, area under the curve >95%. Feature importance was highest for internet regularity and screen status–normalized entropy. The latter correlated most with depressive symptoms (*r* = 0.14, *p* < 0.001). No significant correlation between depression scale and other screen, app or internet connectivity features.	Heterogeneous sample with a majority of males. No clinical diagnosis of depression.	Limited to smartphone-based markers, relatively monomodal. Privacy concerns. Unclear robustness of correlation after including confounders.	N/A	^113^
	Nighttime heart rate variation, regularity of weekday circadian rhythm based on steps (interdaily stability, steps-based daily peaks).	Wrist-worn sensor (Fitbit Charge 2®)	Early validation study, *N* = 267 healthy working adults. 14 consecutive days of remote measurement. Depressive symptoms rated on Patient Health Questionnaire-9.	Accuracy of 80%, sensitivity of 82%, specificity of 78% in predicting depression versus a non-depressed control group. Similar models: - Actigraphy, Beck Depression Inventory (BDI) associates with REM sleep latency and duration, sleep efficiency and fragmentation and sleep duration variability^120^. - Fitbit Charge 2 and 3®, Patient, Health Questionnaire-8 associates primarily with awake time, awakening time, insomnia and mean sleep offset^121^.	Very high interindividual variability, limiting specificity in total population sample and thereby limiting statistical power on group level.	Highly wearable, high compliance.	N/A	^114,120,121^
	Steps, heart rate, motion, light sensor, hours stationary, phone unlocked, screen and app usage, phone calls	Accelerometers and heart rate monitor sensors within smartphone (Android) and smartwatch (Samsung Gear S3®)	Proof of concept study, *N* = 20 with depression. Ecological momentary assessment of different depressive clusters rated by participants.	Accuracy of 96% versus ecological momentary assessment. Sleep and physical activity are most accurate predictors.	Small sample size, very early pilot study.	Requires multiple body-worn sensors, potential compliance challenges. Ambient light exposure requires non-obstructed watch by clothing.	N/A	^115^
Hallucinations and delusions	Rest-activity rhythm	Wrist-worn uniaxial accelerometer	Early validation study, *N* = 79 (29 healthy controls, 50 PD, of which 27 hallucinating vs. 23 non-hallucinating), five days of accelerometry measurements.	Hallucinators showed significantly lower interdaily stability (*p* < 0.01), greater activity during nighttime (*p* < 0.05), and reduced relative amplitude compared to non-hallucinators, independent of most clinical factors such as motor fluctuations.	UPDRS-IV consistently correlated with rest-activity rhythm parameters, suggesting that motor fluctuations may account for part of the elevated activity scores.	N/A	N/A	^131^
*Other*								
Olfactory dysfunction	No eligible studies.							
Color vision disturbances	No eligible studies.							

non-motor symptoms and signs are in bold.

*MCI* mild cognitive impairment, *AD* Alzheimer’s disease, *CV* coefficient of variation, *PSG* polysomnography, *LF/HF* low-frequency/high-frequency, *N/A* not applicable.

^a^Experience with serial assessments in trials, for example the responsiveness of biomarker.

An overview of the TRL for both prodromal and manifest PD per non-motor symptom is presented in Table 2. As different non-motor symptoms have different times of onset and progression across the disease course^21^, we additionally indicated the predictive value of each non-motor symptom in early disease in Table 2. Most proposed digital biomarkers were either a proof of concept or early validation study in healthy controls. Biomarkers for the four sleep-related domains (sleep disturbance, rapid eye movement (REM) sleep behavior disorder, excessive daytime sleepiness and fatigue) were furthest developed with TRLs ranging between 4 and 6 for clinically manifest PD and a TRL of 4 for prodromal PD, with fatigue as exception (TRL 2). The TRL of autonomic function-related biomarkers varied between 2 (bladder dysfunction, orthostatic hypotension) and 6 (heart rate variability) for manifest PD, and were consistently lower for prodromal PD, with the highest TRL of 4 for heart rate variability. Biomarkers for GI-related symptoms had a TRL of 3, and markers for cognitive and neuropsychiatric symptoms had a TRL of 3 (for anxiety and hallucinations) or 4 (for both cognitive deficits and depressive symptoms), and this was identical for manifest and prodromal PD. Sleep-related biomarkers, heart rate variability, constipation, cognitive deficits and depressive symptoms had the highest TRL.

**Table 2:** Developmental phase of digital biomarkers for PD non-motor symptoms in the prodromal and manifest phase

Non-motor symptoms of PD	Developmental phase for prodromal PD	Developmental phase for manifest PD	Value as progression marker in early disease
Sleep and fatigue			
Sleep disturbance	TRL 4	TRL 5	++
REM sleep behavior disorder	TRL 4	TRL 5	+++
Excessive daytime sleepiness	TRL 4	TRL 4	++
Fatigue	TRL 2	TRL 2	+−
Autonomic dysfunction			
Pain	TRL 3	TRL 3	−
Reduced heart rate variability	TRL 6	TRL 6	++
Bladder dysfunction	TRL 2	TRL 2	+
Erectile dysfunction	Not available	Not available	−
Low skin impedance	TRL 3	TRL 3	+
Hyperhidrosis	TRL 3	TRL 3	+−
Aberrant skin temperature rhythm	TRL 3	TRL 4	+
Orthostatic hypotension	TRL 2	TRL 2	++
Gastro-intestinal disorders			
Constipation	TRL 3	TRL 4	++
Dysphagia and esophageal dysmotility	TRL 3	TRL 3	+−
Neuropsychiatric features			
Cognitive deficits	TRL 4	TRL 4	++
Anxiety	TRL 3	TRL 3	+
Depressive symptoms	TRL 4	TRL 4	+++
Hallucinations and delusions	TRL 3	TRL 3	+
Other			
Olfactory dysfunction	Not available	Not available	++
Color vision disturbances	Not available	Not available	−−

TRL of 4 and lower indicates that study populations were not prodromal or manifest PD, or that sensor is only tested in the target population in a lab setting.

The estimation of the potential as PD progression biomarker in trials is the predictive value of the non-motor symptom across the disease course.

### Synthesis of results

A top 3 of the digital biomarkers per non-motor symptom is presented in Table 1. In the Supplementary Table 2, the remaining selected articles per non-motor symptom are listed.

### Critical appraisal of digital biomarkers for non-motor PD symptoms

#### Sleep disturbance

Out of 97 eligible studies, seven studies that assessed sleep using passive digital biomarkers were included^22–28^. Five of these included people with PD, four of which had a sample size >200, and two (proof-of-concept) studies included healthy individuals. Most studies used wrist accelerometry signals as part of a wrist-worn multimodal device. Two experimental studies tested a smart pillow that integrates a skin temperature and sweat sensor with sleep features^28^, and a smart mattress that integrates heart rate variability and respiration sensors with in-bed movement sensors^27^. The following sleep-related features were investigated: immobile bouts, sleep fragmentation (estimated by movement sensors or heart rate variability), and rest-activity circadian rhythm.

With regard to accelerometry, immobility during sleep could predict abnormal polysomnography (the gold standard) with sensitivity and specificity ≥80%^22^. No data was available on the sensitivity to longitudinal disease progression. However, one study reports an association between a composite (digital) sleep score and disease duration in 44 individuals with PD (~1-point decrease per disease year relative to healthy controls score of 13)^22^. One different accelerometry study (*n* = 48) reports that early PD could be distinguished from healthy controls by measuring turning duration and velocity during sleep (3.2 vs. 1.9 s and 16.2 vs 23.4 degrees/second, *p* ≤ 0.001)^24^. With regard to the experimental studies, the mattress-integrated sensor system reports promising heartrate tracking by measuring RR intervals (r^2^ = 0.99, versus ECG)^27^, but reports no performance metrics for estimating sleep parameters. Similarly, conceptual testing of a pillow-integrated sensor system suggests sensitivity to changes in sweat rate and temperature overnight in healthy individuals (no statistics)^28^. TRL ranges between 2 and 6 due to the lack of (longitudinal) data in free-living conditions and uptake as exploratory outcomes in trials. Still, two of the included digital biomarkers are available and approved by regulatory agencies as commercial products, but not for sleep monitoring specifically, limiting TRL to 5 for manifest PD^22,23^.

#### REM sleep behavior disorder

Eight studies measured REM sleep behavior disorder. Three studies included people with RBD (*n* = 35–97), and five studies included people with manifest PD and RBD (*n* = 22–70). All studies had a control group of either healthy controls or individuals with non-RBD sleep disorders. RBD was measured by quantifying nightly movement distribution, wake bouts, immobile bouts and rest-activity cycles, all using wrist actigraphy^29–36^.

An algorithm that measures movement distribution has >90% accuracy in diagnosing RBD in people with PD in free-living conditions^33^. Two other sensors that measure rest-activity cycles and immobile bouts are hampered by low sensitivity (~60%)^29,30^ despite high (~90%) specificity. One study demonstrates increased sensitivity by adding an RBD questionnaire^31^. Studies are short-term and have not explored differences between disease severity levels. Furthermore, sensitivity to disease progression has not been investigated. Three sensors are available for use in research. As studies have not yet deployed such sensors as secondary outcomes in trials, TRL is 5^29,30,33^.

#### Excessive daytime sleepiness

From thirteen eligible studies, seven studies were included^25,26,37–41^. Five studies included people with PD (*n* = 28–106, one observational study with *n* = 239 PD^26^), one large observational study included elderly (*n* = 2920) and one experimental in-lab study included healthy controls (*n* = 30). Excessive daytime sleepiness was defined as immobility during daytime as measured by actigraphy, apart from one experimental study that measured low-frequency/high-frequency (LF/HF) ratio using wrist-worn photoplethysmography.

Concordance for immobile bout detection suggestive of sleep between actigraphy and polysomnography was 85%, mostly due to immobility detection by actigraphy not detected by polysomnography^38^. A large observational study demonstrated that daytime sleeping (napping) >1 h was predictive of PD at least two years prior to diagnosis (odds ratio 1.96, 95% CI 1.25–3.08), whereas the gold-standard Epworth Sleepiness Scale (ESS) was not. If daytime napping was combined with a positive ESS, the odds for later-onset PD further increased (2.52, 95% CI 1.21–5.27)^40^. However, sensitivity to disease progression was not investigated, although one early study suggests no progression in number of daytime naps^25^. LF/HF ratio as a measure of daytime drowsiness levels in an in-lab study showed promising accuracy of 92% for estimating LF/HF ratio from photoplethysmography, but was not tested in free-living conditions. Due to the lack of a large validation trial, TRL remains 4.

#### Fatigue

Four out of nineteen eligible studies measuring fatigue were included^42–45^. All studies included healthy individuals. Three small studies (*n* = 3–35) proxied fatigue by assessing frequency- and time-domain parameters from RR intervals, including low-frequency amplitude and total spectral power). HRV parameters were occasionally combined with other autonomic features, such as respiration rate or skin impedance.

One proof-of-concept used short recordings from smartphone-measured gaze to estimate fatigue levels. Studies only consisted of in-lab proof-of-concept tests on a small number of controls, demonstrating moderate to high accuracy in lab conditions (75–85%). Algorithms have not been assessed in real-world uncontrolled settings and not in the PD population, limiting TRL to 2.

#### Pain

Three of seven eligible studies were included that measured pain passively^46–48^. Pain has only been investigated in controlled conditions among postoperative patients, people with dementia and individuals with human immunodeficiency virus (HIV). Studies were small to moderate in size (*n* = 20–68). Proxies for pain include skin impedance, a combination of psychomotor activity patterns and sleep parameters, and facial micro-expressions. The ground truth used to quantify pain differed from ordinal five-point scales to validated pain scales.

In controlled conditions (at rest or low-intensity activities including walking, sitting, coughing), accuracy for passive pain detection using skin impedance^46^ and circadian motor patterns^48^ ranged between 60–86%, depending on pain severity. One mobile application that measures facial micro-expressions using a smartphone cameras had a high correlation (*r* = 0.88) with a gold-standard pain scale, if combined with questionnaires in the mobile application. Due to early proof-of-concept phase of studies, TRL was limited to 3.

#### Reduced heart rate variability

79 studies were eligible, of which fifteen were included^49–63^. One study included people with PD (*n* = 47), and the other studies (*n* = 5–74) included healthy individuals, and sometimes elderly specifically. Heart rate variability was measured using photoplethysmography in a wide range of different measurement locations, including on chest straps, as part of wrist-worn multimodal smartwatches (i.e. containing additional sensors, mostly accelerometry), unimodal wrist-worn sensors or finger-worn sensors, and one sensor integrated into a smart T-shirt^63^. Most sensors were tested in controlled conditions over short follow-up durations up to a few weeks, in resting states and during exercise. No studies longitudinally investigated sensitivity to disease progression.

Accuracy and error rates of various commercially available sensors against gold-standard (electrocardiogram) show wide differences between devices of similar modality (e.g. wrist-worn, finger-worn, chest-worn). For example, smartwatches demonstrated a large difference between controlled and free-living conditions, with error rates for time domain parameters such as root mean square of the successive differences between RR intervals (RMSSD) between 4 and 13% against the gold standard^52–55^. During the night, sensors appear to have lowest error rates for RMSSDD, likely due to the lower prevalence of motion artifacts^62^. One observational pilot study in PD demonstrated differences in several frequency analysis parameters from RR intervals, including the high-frequency (HF) power, total frequency (TF) power and the low-frequency/high-frequency power (LF/HF) ratio between early PD, advanced PD and healthy controls^56^. As longitudinal studies and a first clinical trial in prodromal PD include heart rate variability as outcomes, TRL is 6^64,65^.

#### Bladder dysfunction

From 20 eligible studies, four small studies (*n* < 50) were included that described passive and non-invasive bladder dysfunction measures by a smart toilet, lower abdomen near-infrared sensor and smartphone microphone^66–69^. Most studies were proof-of-concept tests in healthy controls, and one study included individuals with overactive bladder or outlet obstructions.

In a toilet-integrated sensor system, computer-vision uroflowmeter correlated well with standard uroflowmetry for urination duration and volume (*r* = 0.92–0.96)^66^. Smartphone microphones correlated well with flow rate and volume ( > 0.90), although this was only tested in men^68,69^. Due to the limited level of validation, TRL was 2.

#### Erectile dysfunction

No eligible digital biomarkers have been reported for erectile dysfunction.

#### Low skin impedance

Of 22 eligible studies, four studies measuring skin impedance were included^46,54,58,60^. All studies included healthy controls (*n* = 72 in total). Skin impedance was measured by a wrist-worn or finger-worn sensor, apart from one study investigating a smart vest that integrates multiple physiological parameters^60^.

Overall, the studies lacked extensive performance testing, except for one study which showed high agreement between a wrist-worn smartwatch and a gold standard (without reporting performance metrics). Furthermore, the same device could accurately predict pain severity, strengthening its construct validity as a proxy for pain (accuracy 86%). Measuring skin impedance passively with a wearable device is difficult because movements hinder stable contact with the electrodes and cause noisy data. Yet, wristbands are a feasible option with reasonable measurement accuracy for everyday use. Most studies focused on in-lab testing, but we lack findings regarding the sensitivity of skin impedance measures to longitudinal disease progression. The TRL for these sensors is therefore 3.

#### Hyperhidrosis

From 25 eligible studies, four studies were included^70–73^. One study was an early-validation test in athletes (*n* = 312), and three studies were proof of concepts in healthy individuals (*n* < 5). Sweat rate was measured using humidity sensors on skin patches, an ear-worn impedance sensor or a wrist-worn sensor. One patch saves data locally, the other patch necessitates taking a photo of the patch for colorimetric analysis.

Sweat rate as measured by wearable patches correlated well with gold-standard sensors (hygrometry) in-lab and during controlled exercise or resting conditions in healthy controls (*r* > 0.80). However, most patches are validated in the sports context, skin placement is occasionally difficult and most sensors are not suitable for long-term measurement, limiting TRL to 3.

#### Aberrant skin temperature rhythm

From 22 eligible studies, four studies were included that measured skin temperature passively in healthy controls (*n* = 57 in total) and people with PD (*n* = 12)^74–77^. Temperature sensors were either integrated into a commercialized smartwatch or finger-worn sensor, or a separate patch.

The patches slightly underestimate the skin temperature (~0.6 degrees Celsius), but show a high mean accuracy across various hot/cold settings (accuracy −0.09 degrees Celsius; precision 0.05 degrees Celsius). The finger-worn sensor is still in an early developmental stage, but the smartwatch is already being applied as outcome measure. This smartwatch study suggests an altered circadian skin temperature pattern in individuals with manifest PD^75^. However, the accuracy and longitudinal sensitivity to measure skin temperature rhythm in free-living conditions for manifest PD are unclear, limiting the TRL for these sensors to 4.

#### Orthostatic hypotension

Seven out of fourteen eligible studies that passively measured blood pressure were included^60,78–83^. These studies included healthy controls. Sample sizes ranged considerably, including two smaller studies (*n* = 10 and 25), two medium-sized studies (*n* = 91 and 97) and one large study (*n* = 1057). Blood pressure was measured using a cuffless wrist or finger sensor or a smart T-shirt that integrates a cuff-based sensor with other physiological parameters.

The cuffless sensors showed considerable correspondence to gold standard measurements, with mean differences of −0.1 and 0.0 (SD 3.5–3.6) mmHg for diastolic blood pressure (correlation with gold standard >0.86 across studies). This mean difference is larger in lying and standing position (−0.62 mmHg). The finger worn sensor achieved similar performance metrics with a mean error of 0.9 mmHg systolic and −3.2 mmHg diastolic. The smart vest is still in an early developmental phase but the descriptive results show promising face validity. Cuffless sensors are less intrusive, but are hampered by accuracy of placement and movement artifacts. Whether this is problematic for studies over longer follow-up durations has not been studied. Furthermore, none of the studies have measured sensitivity to drop in blood pressure after standing up, which is necessary for quantifying orthostatic hypotension. Therefore, the digital biomarkers in this category are in TRL 2.

#### Constipation

Five out of twelve eligible studies were included that measured constipation^66,84–87^. These studies included 174 people with PD, 57 healthy controls and 7 people with chronic diarrhea. Three studies measured constipation by an ingestible smart pill, one by measuring abdominal electrodermal activity and one by analyzing excreta through a smart toilet system.

Measurements of the smart pill show construct validity, as transit times correlated with constipation severity (*r* = 0.32) and gold standard scintigraphy (*r* = 0.95). The study investigating the abdominal electrodermal patch reported no test performance metrics, but showed face validity using descriptive results. The smart toilet system is still in an early developmental phase, but stool classification agreed considerably with expert opinion (area under the curve >0.89). Taken together, the sensors show good test performance even in real-life settings. However, the association between sensor based constipation measures and PD disease severity or progression has yet to be investigated. Therefore, the TRL is at 4.

#### Dysphagia

Of 23 eligible studies, five were included^88–92^. In total, they tested 28 people with PD, 97 healthy controls and 80 people with dysphagia or who were being tested for dysphagia.

One device measured dysphagia directly through surface-electromyography of the swallowing muscles, showing a better signal-to-noise ratio compared to conventional electromyography patches. Another device recorded swallowing sounds through a neck-worn microphone, showing a sensitivity of 93.9% to detect a swallow. A submental patch in combination with a mechano-acoustic chest sensor showed a sensitivity of 80% and specificity of 60% to classify dysphagia. Other approaches include a laryngeal displacement sensor, which could distinguish people with PD from healthy controls by their longer swallow duration (*p* < .05) and an intra-oral pressure sensor molded in a mouthpiece (93% accuracy to detect a swallow). Although the proposed sensors perform adequately in controlled environments, submental sensors are quite intrusive to wear for longer durations and. Furthermore, the current sensors have not been tested for their sensitivity to track dysphagia progression. Therefore, TRL was 3 for these biomarkers.

#### Cognitive deficits

From 51 eligible studies, we included eighteen of which we discuss the most advanced ones here^93–109^. Studies were large cohorts of elderly (total *n* > 4000) with one cohort including a subgroup of people with manifest PD. Cognitive deficits have been proxied by various modalities. Most studies deployed wrist accelerometry, for example to track rest-activity rhythm, four studies investigated smart homes containing activity and movement sensors in multiple rooms, and one study deployed smartphone keystroke as a proxy for cognitive dysfunction. Seven larger studies (most *n* > 1000), of which five deployed accelerometry and two a smart home, had longitudinal follow-up up to five years, and demonstrated sensitivity to progression towards MCI or dementia^93–98^.

One smart home study demonstrated an area under the curve of 0.77 for cyclomatic daily activity complexity^99^, while accelerometry studies report hazard ratios for developing dementia of 1.39 for lower rest-activity rhythm in healthy controls and 1.97 for high intra-day activity variability in mild cognitive impairment. Although most sensors are in an early to late validation stage and have not been tested in PD, especially circadian rhythm and walking features show promising performance in cross-sectionally and longitudinally predicting cognitive decline over a years-long prodromal period. The only study including people with PD demonstrated an association between nocturnal sleep disturbance and cognitive function (working memory *r* = 0.28; verbal memory *r* = 0.23), although it is unclear whether cognitive deficits disturb sleep or that this association reflects co-occurrence of symptoms^100^. Taken together, biomarkers for cognitive function are being extensively validated yet focus more on the general than the PD-specific population. The TRL for measuring cognitive deficits using sensors is therefore 4.

#### Anxiety

Three out of thirteen eligible studies were included, encompassing 120 healthy controls and 265 people with anxiety or panic disorders^110–112^. Anxiety was measured using smartphone-recorded speech, a wrist-sensor tracking accelerometry and skin and cardiac autonomic features, or wrist-worn actigraphy monitoring the wake-sleep rhythm. Studies were highly heterogeneous in follow-up duration and measured modality of anxiety, ranging from acute anxiety to progression of generalized anxiety over 18 years.

In algorithmically classified smartphone-recorded speech, higher levels of generalized anxiety were associated with the use of fewer reward-related words (*r* = −0.29). Another study showed that the skin and cardiac autonomic features could accurately detect (acute) psychological stress in 87.2% of the instances, including sitting, running and biking. Lastly, actigraphy was leveraged to track the wake-sleep rhythm, demonstrating predictive value for deterioration of anxiety over an up-to-18 year period (area under the curve = 0.70, sensitivity 84.6%, specificity 52.7%, accuracy 68.7%). The deployed sensors are wearable and feasible to use in everyday life, except for continuous microphone recordings of speech which can be intrusive for privacy reasons. Two studies are in an early developmental stage, whereas one study included longitudinal data. Due to the lack of PD-specific data, TRL for these sensors is 3.

#### Depressive symptoms

64 articles studied the measurement of depressive symptoms using digital biomarkers, of which eighteen were included^113–130^. We discuss the most relevant ones here. Studies mostly included healthy controls (total *n* > 1200). One study included individuals with depressive symptoms (*n* = 20) but no study included people with PD. Depression was usually defined by a standardized depression scale or ecological momentary assessment. Studies typically proxied depressive symptoms with smartphone use data (such as screen time and frequency of use) and smartwatches or wrist-worn sensors that contain photoplethysmography to track heart rate, accelerometers to track activity and rest-activity rhythm, or a combination of these sensors.

Overall, accuracy and sensitivity are good in proof-of-concept observational studies (≥80%), but study follow-up did usually not exceed months, apart from one study that had a 10-week follow-up. Predictive accuracy of depressive symptoms was highest for integrated physiological and both physical and smartphone activity (>80%)^114,115^, although one study in a relatively young heterogeneous population of controls (most <64 years) also reached an accuracy of 96% with smartphone data only against the Patient Health Questionnaire-8^113^. Despite extensive and promising early validation results, there is a lack of longitudinal and PD-specific studies, limiting TRL to 4.

#### Hallucinations and delusions

One observational pilot study in 79 people (27 hallucinating and 23 non-hallucinating people with PD, 29 controls) investigated rest-activity rhythm using a wrist-worn accelerometer.

The study demonstrates lower interdaily stability, greater night-time activity and reduced relative amplitude of activity in hallucinators (*p* < .05), also after correction for clinical status. This observational study lacks large-scale validation and longitudinal testing, but forms an important first step to distill proxies for hallucinations in PD (TRL 3)^131^.

#### Other non-motor symptoms

For olfactory dysfunction and color vision disturbances, no passive digital biomarkers are available or in development.

### General feasibility of digital biomarkers in clinical trials

Multiple studies investigated the feasibility of deploying digital biomarkers with short durations of follow-up in PD, or in populations with other neurodegenerative diseases^132–134^. Populations under investigation included clinically manifest PD (including early stages of PD), other neurodegenerative disorders (e.g. Alzheimer’s disease) and healthy elderly. There were no longitudinal studies that investigated the feasibility in individuals with prodromal disease. Usually, single sensors were investigated, of which wrist accelerometry and smartphone apps were the most common ones. Occasionally, the feasibility of multiple digital biomarkers was assessed. For example, individuals at risk of Alzheimer’s disease wore an Integrated multimodal wrist-worn heart rate variability and accelerometry sensor and had a positive attitude towards current and continued use^135^. One study investigated the short-term compliance and wearability of up to five sensors concomitantly in people with a variety of manifest neurodegenerative diseases, including PD. 98% wore at least three of five sensors throughout a one-week period. Compliance was lower during the night and already reduced at the end of this week^132^.

Various studies investigated longer-term compliance in manifest PD. For example, 13-week compliance (median percentage of data collection) of a combination of a smartphone (app) and smartwatch (accelerometer) that aimed to collect digital signals 24/7 was 68% (mean data collection 16.3 h per day). Supported by a helpdesk and participant feedback when data collection declined, data collection had decreased by 23% after 13 weeks^136^. One large study conducted in nearly 400 people with early PD demonstrated a median smartwatch wear time of 21.1 h per day, with a 5.4% drop-out^7^. Of note, the high compliance was achieved using a multifaceted approach to promote participant engagement, including a helpdesk, personal assessor, newsletters and participant events^64,137^. After a six-month trial of passive smartphone data collection, nearly all people with early PD had a positive attitude towards longer use^138^. With regard to innovative approaches of digital biomarkers, a pilot study of passive in-home sensor monitoring over multiple months was also generally feasible in aging military veterans^139^.

Positive predictors for better compliance were a seamless-fit design, comfortable wear and either a completely unobtrusive and passive design or a clear added benefit to the participant’s daily life in elderly. For a variety of commercially available devices included in the current study, multiple requirements were not met^133^. For example, although wrist-worn sensors were generally considered easiest-to-use^133,134^, limited battery capacity and battery life decline is accompanied by burdensome daily charging and the inability to wear the sensor throughout the day or night. Regarding added benefit for participants, the attitude towards feedback of personal health or activity data is variable and depends on the specific population, disease status and the modality that is measured. For example, compliance with passive digital monitoring of mental health was low over three months in middle-aged veterans^140^.

## Discussion

This study provides an overview of the development of digital biomarkers to measure the presence, severity and rate of progression of non-motor symptoms of PD. We demonstrate that various sleep-related digital biomarkers have been validated in clinically manifest PD, and that biomarkers for REM sleep behavior disorder and excessive daytime sleepiness have been validated in prodromal and at-risk populations. Heart rate variability in particular can be measured passively with high accuracy and likely correlates with disease progression, and promising developments for mood and cognitive features are ongoing in non-PD research fields. For example, By contrast, for several other common non-motor symptoms of PD, the development of passive digital biomarkers is still in an early stage. Specifically, several biomarkers for neuropsychiatric symptoms, autonomic dysfunction and gastro-intestinal features have been developed and tested in healthy participants and patient populations with a neurodegenerative disorder, but these have not yet been validated externally and have uncertain sensitivity to disease progression in PD. In the next sections, we discuss the leading, most promising and least feasible digital biomarkers for PD non-motor symptoms.

### Digital biomarkers with high potential

The most promising biomarkers for prodromal PD are currently in the domain of sleep-related symptoms. Digital biomarkers for REM sleep behavior disorder and excessive daytime sleepiness have already been validated in prodromal cohorts. REM sleep behavior disorder and excessive daytime sleepiness are both predictors for later conversion to manifest PD, making these suitable as selection or progression biomarkers for disease modifying trials^141^. Furthermore, accelerometry-based sleep characteristics such as decreased sleep efficiency and turn velocity have been validated in multiple PD cohorts and correlate well with gold-standard polysomnography. Such features are predictive of PD onset multiple years before diagnosis^142^ and likely remain predictive of progression throughout the disease course^143,144^. Therefore, a continued development, longitudinal validation and feasibility assessment of digital markers for sleep, REM sleep behavior disorder and excessive daytime sleepiness in real-world situations could be helpful to monitor disease progression in prodromal disease-modifying trials. Besides sleep-related symptoms, biomarker development for heart rate variability is in the highest developmental stage of all symptoms. At present, studies indicate that heart rate variability is already disturbed in people with REM sleep behavior disorder^145,146^, reliably distinguishes PD from non-PD^147^ and is cross-sectionally associated with disease severity^148^. However, the sensitivity to disease progression is uncertain. Still, we recommend that future trials that recruit a prodromal or manifest PD population could consider including heart rate variability as exploratory digital progression outcome, given the low measurement burden and the option to capture progression of autonomic dysfunction throughout the course of PD.

### Opportunities for digital biomarkers

Digital biomarkers with high potential but with a limited current developmental stage include neuropsychiatric features, cognitive function, constipation and orthostatic hypotension. These non-motor symptoms have relatively high predictive value in prodromal PD^141,149^ and throughout the disease course^150,151^, although the course of progression of orthostatic hypotension is not well-understood^152^. We therefore consider it likely that in addition to sleep-related features and heart rate variability, these non-motor symptoms will have high value in composite disease progression scores. A recent review outlines perspectives for deploying wearable technology in monitoring depression^153^. Measurement techniques for constipation and orthostatic hypotension are objective and direct, and primarily require validation in PD cohorts. As an alternative to passive proxies of neuropsychiatric features and cognitive function that are currently under development, sensitive active alternatives are already available. For example, widespread use of mobile phones enables frequent text analysis of brief digital questionnaires^154^ and recording and analysis of voice samples to screen for depressive symptoms^155^. Active alternatives to measure cognitive impairment include remote speech assessments^156^ and gait tests using accelerometry sensors^157^. Active alternatives are currently commonly deployed as digital biomarker solution in recent disease modification trials^158^ and have shown good longitudinal feasibility in PD^138^. Several initiatives for further development of passive digital biomarkers for neuropsychiatric features are currently underway, such as digital deep phenotyping initiatives^159,160^.

For markers of autonomic dysfunction, many proposed sensors were poorly integrated for use in PD trials. For example, for hyperhidrosis monitoring, a combination of an accurate patch^70^, remote synchronization^71^ and longer measurement duration possibilities^72^ is necessary for passive monitoring opportunities. From a technological point of view, the development of unobtrusive and specific passive markers for dysphagia (although primarily a motor symptom), hallucinations, pain and bladder dysfunction is most challenging, despite some of these having high value in predicting disease progression^161–163^.

Passive digital biomarkers were not available at all for color vision disturbances, erectile dysfunction and olfactory dysfunction. However, well-validated active or self-administered tests are available for erectile dysfunction and olfactory dysfunction^164,165^, and under development for color vision disturbances^166,167^. Recent evidence suggests that olfactory dysfunction might be predictive of symptom decline or a more malignant PD phenotype^168^ and that non-invasively measuring olfactory bulb function directly shows promise as a diagnostic marker and possibly as a progression marker^169^. Further development of remote active digital tests for these non-motor symptoms is likely the most rational way forward.

### Ethical considerations

There are several ethical considerations regarding widespread digital biomarker development for and deployment in clinical trials. First, to what extent novel digital biomarkers should be patient-centric and patient-relevant compared to more sensitive but potentially clinically irrelevant markers is currently debated^170,171^. The American Food and Drug Administration (FDA) has recently released guidance documents on patient-focused drug development and argues for a combination of sensitive markers that directly reflect patient-relevant outcomes. This guideline followed on the public debate about the designation of expensive orphan drug status based on outcome measures without direct clinical relevance^16^. Including patients in the development and evaluation of new biomarkers is often overlooked, which hampers long-term implementation for various reasons^172^. In this study, people with PD were part of the assessment to give better insight in longer-term feasibility, including relevance of measurement, participant burden and limitations of use, which is essential in development of novel digital applications^173,174^. Lastly, data ownership and privacy issues require a solid debate, especially with digital biomarkers of non-motor symptoms, intrusive passive markers monitoring daily activity, mobile phone typing and speech as well as geographical and sometimes even semantic location. This subject is outside the scope of this review and has been carefully reviewed elsewhere^175^.

### Limitations

This study has several limitations. Despite the existence of various roadmaps^172^ and frameworks^171,176–178^ for the development, analysis and critical appraisal of digital biomarkers, TRL classification of sensors in digital biomarkers is hampered by the high heterogeneity in validation methods and study reporting. Due to this heterogeneity across symptom categories, the complexity of the included research could not be reduced into one validated scale for assessment of validity, reliability or feasibility. In addition, we restricted ourselves to peer-reviewed literature, which does not necessarily reflect either the latest stage of development or commercial availability which can be found through commercial channels or in grey literature. Furthermore, the selection of non-motor symptoms was based on the MDS-NMSS, which does not include PD non-motor symptoms previously unappreciated such as (central) respiratory dysfunction, for which several early-stage digital sensors are under development^179–183^. Lastly, inherent to many non-motor symptoms, objective measurement is often limited to measuring proxies of those symptoms. Notably, similar proxies are deployed for different non-motor symptoms. For example, skin impedance is included both as sign of autonomic dysfunction and as proxy for pain, and psychomotor activity or sleep-wake cycle are leveraged as a proxy for cognitive function, depression, anxiety and hallucinations. This suggests a (current) lack of specificity of such sensors as proxies of non-motor outcomes, and might hamper the interpretation of disease progression. Measuring proxies complicates the translation from both non-PD to PD and from lab studies to real-world situations, in which various known and unknown factors might interfere with those proxies with negative effect on test performance^178^. Combing multiple measurement modalities might alleviate such challenges^184^. Such a composite multi-symptom score has additional merit for improved sensitivity and more consistent predictive power across individuals. A major strength of this study is the comprehensive search strategy expanded beyond the PD-field. This strategy yielded several additional markers for various non-motor symptoms undetected with a previous scoping review that was limited to PD populations^18^. We envision that this broader search gives PD researchers insight in high-potential developments to be translated to their own portfolio or promote interdisciplinary collaboration. Thereby, this study provides a basis for further discussions on the role of digital outcomes in disease modification trials, all of which were still deemed exploratory in a recent consensus paper^185^.

## Conclusion

Here, we have critically reviewed the current state of the art of non-motor digital biomarkers for PD, from inside and outside the PD research field. Furthermore, we have provided insight into the opportunities and challenges for the further development of these important biomarkers. Passive digital sleep-based biomarkers can currently be deployed in clinical trials as secondary outcome, and autonomic biomarkers and neuropsychiatric biomarkers show the highest short-term potential. However, these first require further internal validation before they can be engaged as exploratory outcomes. Future studies may use our critical reflection as a starting point for further digital biomarker development, for subsequent deployment in clinical trials, and ultimately for their application in clinical practice.

## Methods

### Protocol and registration

This study adheres to the PRISMA-ScR guidelines for scoping reviews and uses the PRISMA-S guidelines for reporting the search strategy. The protocol was pre-registered through the Open Science Framework (https://osf.io/ubyc2/).

### Eligibility criteria

All digital biomarkers for non-motor symptoms were considered if the measured modality was objective (not directly influenced by the participant, such as with electronic diaries) and collected data passively. This means that the biomarker should take measurements during natural uninterrupted behavior of individuals in daily life, contrary to active monitoring which requires specific tasks to be performed. Passive measurement was an inclusion criterion due to the level of objectiveness and long-term compliance, important for the feasibility of longitudinal cohorts and clinical trials.

If a potential marker was an indirect proxy of a non-motor symptom, the marker had to be a clear reflection of and closely related to the respective non-motor symptom to be eligible. For example, free-living activity levels were considered as a potential marker of fatigue, but association studies merely reporting co-occurrence of two symptoms were not eligible. Biomarkers could be measured through any modality, irrespective of the shape and location of the measurement application, as long as it was non-invasive. Examples include wearables (e.g. smartwatch, bracelet), furniture-integrated sensors (smart bed, smart toilet), software (mobile phone apps, computer programs), automated video recording and recording of use (computer).

All peer-reviewed articles in English published after 2006 were considered. In addition, we included all conference abstracts published in the last 5 years, which allowed for inclusion of the latest developments. References of included articles were scanned for additional eligible studies. All non-motor symptoms as recognized in the Non-Motor Symptom Scale (NMSS) of the Movement Disorders Society (MDS-NMSS) were considered, including dysphagia (although primarily a motor symptom). Skin impedance and temperature were included as additional putative early signs of autonomic dysfunction^186^.

### Information sources and search

PubMed and EMBASE were initially screened systematically for eligible studies on November 25th, 2021. The search was updated in December 2023.

The structured search string consisted of two elements. The first element included synonyms of all non-motor symptoms or signs, and the second contained a string of synonyms of digital biomarker device modality-related terms. The search strings are made available in the Supplementary Table 1. Because of the large number of articles, and to avoid decision-making bias, the selection process was divided into two stages.

### Stage 1: Selection of sources of evidence based on article scope

Four authors (JJD, RB, EP, MM) independently evaluated the eligible entries. Each entry was assigned to two authors, such that all articles were screened and considered for inclusion twice. First, all articles were screened for title and abstract, after which full-text screening was conducted. The main selection criterion was whether the article met the eligibility criteria (see above). Disagreements were resolved in consensus meetings with these four authors.

From all selected full-texts, we extracted a predefined set of variables in a standardized extraction form. Included variables were first author, title, year of publication, journal, study type and objective (validation study, proof of concept, etc.), the measured non-motor symptom, tested population(s), and main field of development (PD or other).

### Stage 2: Selection of sources of evidence based on relevance

Based on all selected full-text articles, the same four authors independently selected a top 5 for each non-motor symptom presenting the most advanced state of the field, to reduce the large body of developments into a clear overview of the state of the art, which is the main goal of this study. The selection was based on 1) methodological rigor of the study design (sample size, repeated measures, comparisons made), 2) test performance (validity and reliability) and 3) strength of the evidence for the association between the measured modality and the non-motor symptom (when the digital marker was an indirect proxy). Due to the high heterogeneity in symptoms, study design and reported test parameters within each symptom category, we argued there was no universal scale suitable for assessing such heterogeneous parameters across symptom categories. Therefore, instead, studies were evaluated within each symptom category based on the pre-defined set of three parameters. Any discrepancies in the selection were discussed in multiple meetings by the four authors until consensus was reached.

For the top 5 articles, data extraction was extended by additionally collecting the sensor or system name, the device used, the modality (direct measurement of clinical sign, surrogate marker?), the test setting (clinic, lab or home, free-living), and data on the validity, reliability and feasibility (see below) of this biomarker. We paid particular attention to longitudinal studies reporting serial biomarker measurements, as this gives insight in the sensitivity of a biomarker as disease progression biomarker.

### Stage 3: Critical appraisal and analysis

In multiple author meetings, critical appraisals were conducted for the proposed top 5 of digital biomarkers per non-motor symptom. In these sessions, validity, reliability, and feasibility of all biomarkers was evaluated by members with clinical expertise in prodromal and manifest PD, as well as with expertise in sensor development, validation and application of technology in PD. Three patient researchers (RvdH, HM, SM) were part of the critical appraisal process to voice the patient experience with special regard to feasibility.

We evaluated the following variables:

- Validity of the biomarker versus the (gold-)standard, with a specific focus on discriminant and criterion validity.
- Reliability, based on reported test-retest reliability.
- Feasibility, based on the following subdomains: user friendliness (including stigmatization), compliance, implementation and practicality (how easily is it deployed in the participant’s context), based on both quantitative variables and qualitative interpretation. This analysis was conducted together with patient researchers, informed by a roadmap for implementation of patient-centered digital outcomes^172^.
- Sensitivity to disease progression, as measured by serial measurements in a longitudinal study, if applicable.

**Fig. 2:**
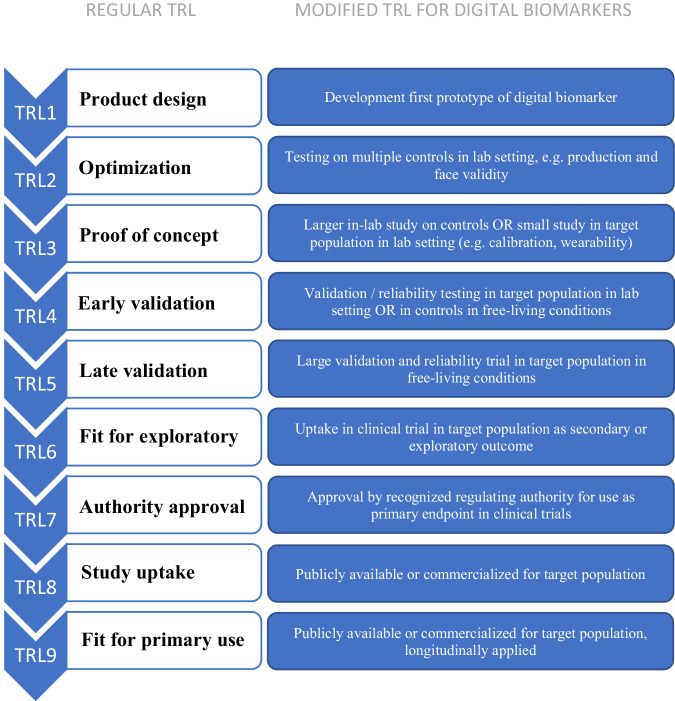
Technological readiness level (TRL)^190^ for digital biomarkers in clinical trials.

Based on these results, a grade for the stage of development within a specific symptom category was made. The stage of development was based on the Technological Readiness Level (TRL), as defined by the most recent European Union definitions which we adapted to fit the field of digital biomarkers and medical monitoring devices in general (Fig. 2)^187^. Developmental stages range from development or proof of concept (TRL1, lowest), to public availability or commercialization applied in a longitudinal study (TRL9, highest) via several early and late validation stages in either lab setting or free-living conditions. We assigned a TRL per non-motor symptom or sign separately for the prodromal phase and for the clinically manifest phase. Finally, using a structured process in which both performance metrics, TRL and applicability in PD were assessed, all authors agreed on a final top 3 of digital biomarkers per non-motor symptom to be presented in one main table. The remainder of the included studies was added to Supplementary Table 2.

### Supplementary information

Supplementary materials

## Data Availability

All data generated or analyzed during this study are included in this published article and its supplementary materials. A reference manager file is available from the authors.
