# Supplementary material for: Digital biomarkers for non-motor symptoms in Parkinson’s disease: the state of the art
Source: NPJ Digit Med. 2024 Jul 11;7:186. doi: 10.1038/s41746-024-01144-2 (PMC11239921; doi:10.1038/s41746-024-01144-2)
Supplement: Supplementary file 1 — Supplementary materials [file 41746_2024_1144_MOESM1_ESM.pdf]

Supplementary Table 1: Search strategy

PubMed search strategy

("REM Sleep Behavior Disorder"[MeSH Terms] OR "RBD"[Title/Abstract] OR "parasomn*"[Title/Abstract] OR "Disorders of Excessive Somnolence"[MeSH Terms] OR "somnolen*"[Title/Abstract] OR "hypersomn*"[Title/Abstract] OR "sleepiness"[Title/Abstract] OR "drowsiness"[Title/Abstract] OR "fatigue"[MeSH Terms] OR "fatigue*"[Title/Abstract] OR "tired*"[Title/Abstract] OR "weariness"[Title/Abstract] OR "lassitude"[Title/Abstract] OR "lack of energy"[Title/Abstract] OR "energy lack"[Title/Abstract] OR "lacking energy" [Title/Abstract] OR "pain"[MeSH Terms] OR "pain"[Title/Abstract] OR "cramp"[Title/Abstract] OR "cramps"[Title/Abstract] OR "ache"[Title/Abstract] OR "aches"[Title/Abstract] OR "Hyperhidrosis"[MeSH Terms] OR "Hyperhidrosis"[Title/Abstract] OR "sweat*"[Title/Abstract] OR "perspiration"[Title/Abstract] OR "transpiration"[Title/Abstract] OR "Sialorrhea"[MeSH Terms] OR "Sialorrhea"[Title/Abstract] OR "drool*"[Title/Abstract] OR "hypersalivation"[Title/Abstract] OR "hyper-salivation"[Title/Abstract] OR "saliva*"[Title/Abstract] OR "heartrate variability"[Title/Abstract] OR "heart-rate variability"[Title/Abstract] OR "heart rate variability"[Title/Abstract] OR "heart-rate regulation"[Title/Abstract] OR "urinary bladder, neurogenic"[MeSH Terms] OR "urinary bladder, underactive"[MeSH Terms] OR "urinary bladder, overactive"[MeSH Terms] OR "Urination Disorders"[MeSH Terms] OR "Nocturia"[Mesh] OR "bladder"[Title/Abstract] OR "urination"[MeSH Terms] OR "detrusor"[Title/Abstract] OR "urinary dysfunction"[Title/Abstract] OR "urinary tract dysfunction"[Title/Abstract] OR "Erectile Dysfunction"[MeSH Terms] OR "impoten*"[Title/Abstract] OR "erecti*"[Title/Abstract] OR "Electric Impedance"[MeSH Terms] OR "skin conductance" [Title/Abstract] OR "skin impedance"[Title/Abstract] OR "Skin Temperature"[MeSH Terms] OR "Skin Temperature"[Title/Abstract] OR "hypotension, orthostatic"[MeSH Terms] OR "orthostatic hypotension"[Title/Abstract] OR "postural hypotension"[Title/Abstract] OR "constipation"[MeSH Terms] OR "constipat*"[Title/Abstract] OR "gut motility"[Title/Abstract] OR "stool*"[Title/Abstract] OR "Dyschezia"[Title/Abstract] OR "Deglutition Disorders"[MeSH Terms] OR "dysphagia"[Title/Abstract] OR "esophageal dysmotil*"[Title/Abstract] OR "oesophageal dysmotil*"[Title/Abstract] OR "swallow*"[Title/Abstract] OR "peristalsis"[Title/Abstract] OR "Cognitive Dysfunction"[MeSH Terms] OR "cognitive impairment*"[Title/Abstract] OR "neurocogniti*"[Title/Abstract] OR "cognitive decline"[Title/Abstract] OR "mental deterioration"[Title/Abstract] OR "anxiety"[MeSH Terms] OR "anxiety"[Title/Abstract] OR "nervousness"[Title/Abstract] OR "hypervigilan*"[Title/Abstract] OR "anxious*"[Title/Abstract] OR "Depressive Disorder"[MeSH Terms] OR "depression"[MeSH Terms] OR "Depressive Disorder"[MeSH Terms] OR "depressi*"[Title/Abstract] OR "hallucinations"[MeSH Terms] OR "hallucinat*"[Title/Abstract] OR "Olfaction Disorders"[MeSH Terms] OR "olfact*"[Title/Abstract] OR "hyposmi*"[Title/Abstract] OR "anosmi*"[Title/Abstract] OR "dysosmi*"[Title/Abstract] OR "smell*"[Title/Abstract] OR "Color Vision"[MeSH Terms] OR "Color Vision Defects"[MeSH Terms] OR "Color Vision"[Title/Abstract] OR "colour vision"[Title/Abstract])

AND

("digital biomarker*"[Title/Abstract] OR "portable*"[Title/Abstract] OR "digital technolog*"[Title/Abstract] OR smart technolog*[Title/Abstract] OR digital health

technolog*[Title/Abstract] OR “monitoring system*”[tiab] OR biomonitor*[Title/Abstract] OR “tracking system*”[tiab] OR “tracker*”[Title/Abstract] OR “measurement device*”[Title/Abstract] OR ("sensor*"[Title/Abstract] AND "digital*"[Title/Abstract]) OR ("sensor*"[Title/Abstract] AND "device*"[Title/Abstract]) OR ("digital*"[Title/Abstract] AND "device*"[Title/Abstract]) OR "smart device*"[Title/Abstract] OR “motion sensor*”[Title/Abstract] OR actigraph*[Title/Abstract] OR "inertial sensor*"[Title/Abstract] OR "Cell Phone"[Mesh] OR mobile technolog*[Title/Abstract] OR mobile device*[Title/Abstract] OR "mobile phone*"[Title/Abstract] OR smartphone*[Title/Abstract] OR "smart phone*"[Title/Abstract] OR "Wearable Electronic Devices"[MeSH Terms] OR "wearable*"[Title/Abstract] OR smartwatch*[Title/Abstract] OR smart watch*[Title/Abstract] OR "ingestible"[Title/Abstract]) NOT (“in vitro”[title] OR animal[title] OR rats[title] OR rat[title])

Embase

(parasomnia/ OR RBD.ti,ab,kf. OR parasomn*.ti,ab,kf. OR exp somnolence/ or exp daytime somnolence/ OR somnolen*.ti,ab,kf. OR hypersomn*.ti,ab,kf. OR sleepiness.ti,ab,kf. OR drowsiness.ti,ab,kf. OR fatigue/ or exhaustion/ or lassitude/ OR fatigue*.ti,ab,kf. OR tired*.ti,ab,kf. OR weariness.ti,ab,kf. OR lassitude.ti,ab,kf. OR lack of energy.ti,ab,kf. OR energy lack.ti,ab,kf. OR lacking energy.ti,ab,kf. OR musculoskeletal pain/ or myalgia/ or chronic pain/ OR pain.ti,ab,kf. OR cramp.ti,ab,kf. OR cramps.ti,ab,kf. OR ache.ti,ab,kf. OR aches.ti,ab,kf. OR hyperhidrosis/ OR Hyperhidrosis.ti,ab,kf. OR sweat*.ti,ab,kf. OR perspiration.ti,ab,kf. OR transpiration.ti,ab,kf. OR hypersalivation/ OR Sialorrhea.ti,ab,kf. OR drool*.ti,ab,kf. OR hypersalivation.ti,ab,kf. OR hyper-salivation.ti,ab,kf. OR saliva*.ti,ab,kf. OR heartrate variability.ti,ab,kf. OR heart-rate variability.ti,ab,kf. OR heart rate variability.ti,ab,kf. OR heartrate regulation.ti,ab,kf. OR heart-rate regulation.ti,ab,kf. OR bladder disease/ OR bladder dysfunction/ OR impaired bladder emptying/ OR neurogenic bladder/ OR overactive bladder/ OR micturition disorder/ OR Nocturia/ OR nocturia.ti,ab,kf. OR bladder.ti,ab,kf. OR micturition/ OR detrusor.ti,ab,kf. OR urinary dysfunction.ti,ab,kf. OR urinary tract dysfunction.ti,ab,kf. OR male sexual dysfunction/ or impotence/ or potency disorder/ OR impoten*.ti,ab,kf. OR erecti*.ti,ab,kf. OR skin conductance/ OR skin impedance.ti,ab,kf. OR skin temperature/ OR Skin Temperature.ti,ab,kf. OR orthostatic hypotension/ OR orthostatic hypotension.ti,ab,kf. OR postural hypotension.ti,ab,kf. OR Constipation/ OR constipat*.ti,ab,kf. OR gut motility.ti,ab,kf. OR stool*.ti,ab,kf. OR Dyschezia.ti,ab,kf. OR Dysphagia/ OR dysphagia.ti,ab,kf. OR esophageal dysmotilit*.ti,ab,kf. OR oesophageal dysmotil*.ti,ab,kf. OR swallow*.ti,ab,kf. OR peristalsis.ti,ab,kf. OR cognitive defect/ or mild cognitive impairment/ OR cognitive impairment*.ti,ab,kf. OR neurocogniti*.ti,ab,kf. OR cognitive decline.ti,ab,kf. OR mental deterioration.ti,ab,kf. OR Anxiety/ OR anxiety.ti,ab,kf. OR nervousness.ti,ab,kf. OR hypervigilan*.ti,ab,kf. OR anxious*.ti,ab,kf. OR depression/ OR depressi*.ti,ab,kf. OR hallucination/ or auditory hallucination/ or olfactory hallucination/ or visual hallucination/ OR hallucinat*.ti,ab,kf. OR exp smelling disorder/ OR olfact*.ti,ab,kf. OR hyposmi*.ti,ab,kf. OR anosmi*.ti,ab,kf. OR dysosmi*.ti,ab,kf. OR smell*.ti,ab,kf. OR color vision/ OR color vision defect/ OR Color Vision.ti,ab,kf. OR colour vision.ti,ab,kf.)

AND

(digital biomarker*.ti,ab,kf. OR digital technolog*.ti,ab,kf. OR digital health technolog*.ti,ab,kf. OR portable*.ti,ab,kf. OR measurement device*.ti,ab,kf. OR monitoring system*.ti,ab,kf. OR biomonitor*.ti,ab,kf. OR tracking system*.ti,ab,kf. OR tracker*.ti,ab,kf. OR smart technolog*.ti,ab,kf. OR (sensor*.ti,ab,kf. adj1 digital*.ti,ab,kf.) OR (sensor*.ti,ab,kf. adj1 device*.ti,ab,kf.) OR

(digital*.ti,ab,kf. adj1 device*.ti,ab,kf.) OR smart device*.ti,ab,kf. OR motion sensor*.ti,ab,kf. OR actigraph*.ti,ab,kf. OR inertial sensor*.ti,ab,kf. OR mobile technolog*.ti,ab,kf. OR mobile device*.ti,ab,kf. OR exp mobile phone/ OR smartphone*.ti,ab,kf. OR smart phone*.ti,ab,kf. OR mobile phone*.ti,ab,kf. OR exp wearable computer/ OR wearable*.ti,ab,kf. OR smartwatch*.ti,ab,kf. OR smart watch*.ti,ab,kf. OR ingestible.ti,ab,kf.) NOT (in vitro.ti. OR animal.ti. OR rats.ti. OR rat.ti.)

(1 = full search string)

2: limit 1 to conference abstracts

3: 1 not 2

4: limit 1 to (conference abstracts and yr="2016 -Current")

5: 3 or 4
(6: limit 5 to yr="2006 -current")

Supplementary Table 2: Digital biomarkers of prodromal PD symptoms currently available or under development (maximum of 4 per symptom), and other developments for digital biomarkers of every non-motor symptom, where applicable.

Prodromal symptom	Methods	Validity, reliability and feasibility and longitudinal measurements
**Sleep**		
Bed-based instrumentation interesting different type of sensor[1]	This multicenter interventional study used unobtrusive nighttime monitoring for sleep quality in severely disabled autistic children. totally n=52 students with (age 5-22)	Smart mattress with limited testing (only very limited proof of concept), designed mostly for children with autism in which heart rate, respiration rate and movement can be tracked reliably. The temperature sub-system for enuresis monitoring needs PSG comparisons in future studies.
A Smart Pillow for Health Sensing System Based on Temperature and Humidity Sensors[2]	Proof of concept, this research has conducted an interventional study to investigate the sleep disturbances (sweat, fever, insomnia) of one patient using a smart pillow that monitors the body temperature based on the Fuzzy logic system as well as parameters such as sleep position, duration of sleep and turns.	Proof of concept, one individual has been tested for one night. A smart pillow is developed to monitor body temperature, humidity, sleep position and duration, and turns.<br>Limitation: data recorded every 5 minutes and all data more granular data within 5 min is lost. Limited accuracy, only one night testing in one patient.
**Other developments:**<br>- Nocturnal awakenings and sleep fragmentation were associated with PD progression.[3]<br>- Increased sleep latency and reduced efficiency and REM sleep were associated with PD.[4]		
**RBD**		
Towards a handy screening tool for REM sleep behavior disorder: RDBAct algorithm from wrist actigraphy data[5]	Early validation study of automatic REM sleep without atonia (RSWA) detection. 25 patients with PD (underwent video-PSG with bilateral wrist actigraphy. 31 video recordings of RSWA and 18 without RSWA were used for training and testing.	Wrist actigraphy had AUC of 0.67 in detecting RSWA.
**Other developments:**<br>- Expert-based visual interpretation of actigraphy outperformed quantitative actigraphy analyses.[6]<br>- Low sensitivity (20%) of REM sleep behavior disorder detection by actigraphy can be increased by adding an REM sleep behavior disorder questionnaire.[7]<br>- Number of wake bouts is higher in people with PD with REM sleep behavior disorder compared to people with without REM sleep behavior disorder.[8]		

**Excessive daytime sleepiness (EDS)**		
**Other developments:**<br>- Daytime napping on actigraphy does not correlate with subjective EDS severity as measured by Epworth Sleepiness Scale[9,10]<br>- Excessive daytime sleepiness is associated with shows significant EDS based on MSL[4]<br>- Frequency of daytime naps was associated with PD relative to controls but was not progressive over multi-year follow-up.[3]		
**Fatigue**		
The association between heart rate variability, reaction time, and indicators of workplace fatigue in wildland firefighters[11]	n=10 participants in a 14-day period were examined to investigate the relationship between heart rate variability and incidence of fatigue, total sleep time, and reaction time in shift worker by using wrist actigraphy in laboratory setting.	There is significant association between HRV and incidence of fatigue and sleep. Efficient for monitoring daily cardiac function in response to stressful situation.
Smartphone-based gaze as digital biomarker for mental fatigue[12]	Proof-of-concept, two mental fatigue checking systems were used, consisting of a language-independent, object-tracking Task system was used for 17 patients and proofreading task was used for 15 patients, data collected in lab.	Mental fatigue was predicted with 80% accuracy by smartphone-measured gaze (as a digital biomarker) by using 75-150 sec of gaze data.
**Pain**		
No supplementary studies.		
**Hyperhidrosis**		
Wearable Sweat Rate Sensors for Human Thermal Comfort Monitoring[13]	Proof of concept, 3 healthy subjects with average age of (27.7 +-3.2) using a wrist-worn sensor, human sweat rate measurement in the lab.	Watch-type sensor, 4-5 hours with measurement period of 2 min, the sensor is capable to measure the sweat rate with a linearity of 97.9% and sensitivity of 0.0039 over a range of 3.76 to 137.68 (g/m2h)
**HRV**		
Development of a Novel Wearable Ring-Shaped Biosensor[14]	A Novel Wearable Ring-Shaped was designed for measuring of Galvanic Skin Response (GSR) and Heart Rate Variability (HRV) in n=4 participants	2 out of 3 HRV measurements with the wearable were compatible with commercial HRV sensor know as gold-standard (<10%).
Assessing the Quality of Heart Rate Variability Estimated from Wrist and Finger PPG: A Novel Approach Based on Cross-Mapping Method[15]	n=16 (7 female) healthy subjects (age 28.5 +-3.5) were examined to investigate the non-invasiveness of photoplethysmography (PPG), and pulse rate variability (PRV) signal quality reported from wrists and fingers.	Nonlinear dynamic approach indicated PRVs from wrist and finger both reported a good correlation coefficient with HRV (between 0.80-0.95), finger is easier and resulted to be more reliable
Smart Vest: wearable multi-parameter remote physiological monitoring system[16]	A wearable physiological monitoring system is designed, sends the measured data to a data control station through wireless communication and global positioning system (GPS) modules. Vest measures electrocardiogram (ECG), photoplethysmogram (PPG), body temperature, blood pressure, galvanic skin response (GSR) and heart rate	Proof of concept, an effort to build a technology for remote physiological monitoring, concept is feasible. Reasonable accuracy for measurements (mostly within two standard deviations), however too low accuracy for medical application.

Heart Rate Variability from Wearables: A Comparative Analysis Among Standard ECG, a Smart Shirt and a Wristband[17]	Heart Rate Variability (HRV) was checked in three phase position, including sitting, standing and during breathing by three methods: standard one-lead electrocardiography (ECG), commercial ECG shirt and inter-beat-intervals (IBI) measured by a research-grade photoplethysmographic (PPG) wristband.	Shirt is generally more accurate and reliable compared to wrist band. Accuracy highly dependent on physical characteristics such as wrist and chest size.
A comprehensive accuracy assessment of Samsung smartwatch heart rate and heart rate variability[18]	Samsung Gear Sport smartwatch is compared (n=28) with Shimmer3 ECG device in free-living conditions for 24 hours.	Good correlations with HR and time-domain parameters, whereas LF, HF and other frequency-domain were less consistent and situation-dependent.
**Bladder dysfunction**		
Smartphone App for In-home Uroflowmetry[19]	Correlation between standard uroflowmetry and a smartphone app that analyzed urine voiding sounds to calculate flow rate and volume. 47 individuals with overactive bladder or outlet obstruction, and 15 healthy controls, all performing ≥10 self-measurements.	Excellent correlation between gold standard and smartphone app (Pearson's correlation coefficient 0.91-0.92).
**Erectile dysfunction**		
**Other developments:** In a recent review, a novel framework for the development of state-of-the-art digital biomarkers for erectile dysfunction is proposed. Options include skin temperature, arterial pulse using PPG, radial circumference and rigidity, oxygen saturation.[20]		
**Skin impedance**		
Pain Assessment Tool with Electrodermal Activity for Postoperative Patients: Method Validation Study[21]	n=25 postoperative patients (age 23-89) whit history of moderate to severe pain, wore Empatica E4 wristband during intensity activity. The pain intensity was self-reported with the numeric rating scale (NRS).	GSR data collected easily by using affordable wearable devices. Best fitting model had an accuracy of 86.0%
**Skin temperature**		
Kick Ring LL: A Multi-Sensor Ring Capturing Respiration, Electrocardiogram, Oxygen Saturation, and Skin Temperature[22]	A smart ring implemented with PPG, ECG and Thermistor to detect HR, RR, Spo2 and temperature. Validation against gold standard in-clinic for short period, in n=2 participants.	Proof of concept. Experimentally reported HR & RR had significant correlation with their related standards. But Spo2 had trial-dependent similarities and temperature measurements fell within normal range (correlation up to r=0.85, p<0.05).
**Orthostatic Hypotension**		
Cuffless Blood Pressure Monitoring from an Array of Wrist Bio-Impedance Sensors Using Subject-Specific Regression Models: Proof of Concept[23]	Proof of concept, n=10 healthy participants (age 18-30). Post-exercise BP measurement by using low-noise bio-impedance sensing hardware placed on radial and ulnar arteries of the wrist.	Wrist sensor capable of cuffless BP-monitoring, accuracy: Correlation up to 0.86 for systolic BP and 0.77 for diastolic BP. Better accuracy than two other studies with accuracies as high as +-7 and +-5 mmHg for systolic and diastolic blood pressure respectively.[24,25]

		Sensitivity of orthostatic hypotension measurement not investigated.
Smart Vest: wearable multi-parameter remote physiological monitoring system.[16]	Pilot study, n = 25 (healthy). Smart T-shirt contains ECG and photoplethysmography waveforms. Data of both sensors are transmitted to a remote physiological monitoring station along with the geo-location of the wearer, and analyzed there.	Dynamic BP not yet sufficiently accurate compared to non-invasive BP monitor (risk of underperforming on acute changes in pressure). Focused on military use. User-specific calibration necessary. Not fit for different weather conditions.
**Constipation**		
A mountable toilet system for personalized health monitoring via the analysis of excreta[26]	Proof of concept, both stool, urine analysis and uroflowmetry. Toilet system integrates urinary and gastro-intestinal voiding using both pressure and motion sensors.<br>Urine analysis prediction accuracy measured once in n = 2. Stool analysis prediction accuracy measured in n = 11 over 5 weeks.	Proof of concept, still needs to be finetuned and tested in larger populations. Stool analysis requires bulky and expensive analytical equipment. Urinalysis and uroflowmetry modules demonstrate high accuracy potential, stool analysis has high AUC (>0.89).
Wireless Capsule Motility: Comparison of the SmartPill® GI Monitoring System with Scintigraphy for Measuring Whole Gut Transit[27]	Validation study, N=10 healthy adults. Ingestible capsule containing motion sensors (SmartPill®). Measuring parameters of colonic motility and transit times. Capsule is validated against gold-standard scintigraphy.	Capsule is sensitive to opioid effect on gut transit time.<br>R=0.95 for scintigraphy vs. pill.<br>More convenient and less invasive than scintigraphy.<br>Difficult for individuals with dysphagia.
**Dysphagia**		
An electronic device measuring the frequency of spontaneous swallowing: digital phagometer[28]	n=42 (21 PD and 21 HC). Sensor placed on neck, measuring laryngeal displacement, swallowing frequency, swallowing time.	Longer swallow duration distinguishes PD (n=21) from controls, but unclear whether diagnostic for dysphagia itself. Swallowing frequency potential determinant.
Neural Network Pattern Recognition of Lingual–Palatal Pressure for Automated Detection of Swallow[29]	n=19 (7 PD and 12 HC). Seven Intra-oral pressure sensor placed in molded mouthpiece fitting over the upper teeth and hard palate. Pressure measures during swallow and non-swallow times analyzed using a detection algorithm.	Palatometry signals can detect the oral phase of swallowing with high accuracy. The TDANN could create an algorithm with high sensitivity, specificity (up to accuracy of 96%) and temporal response.
**Cognitive deficits**		
Clustering Home Activity Distributions for Automatic Detection of Mild Cognitive Impairment in Older Adults[30]	85 homes, age>=70, cognitively healthy (MMSE>24, CDR score<0.5) and independent person lives in a larger than a room apartment, passive infrared motion sensors were installed to detect movement and general activity	Mild cognitive impairment detection in older adults with an F0.5 score of 0.856 and indicate non-amnestic sub-type of MCI with F0.5 score of 0.958.
Circadian rest-activity rhythm and longitudinal brain changes underlying late-life cognitive decline[31]	To investigate the longitudinal relationship between circadian rest-activity rhythm alterations and late-life cognitive decline in older adults without dementia, n=129 participants (mean age of 69.3 ± 7.7 years) from the Korean Brain Aging Study for Early Diagnosis and Prediction of Alzheimer's Disease (KBASE) cohort were involved. Baseline and 2-year follow-up assessments included actigraphy, Pittsburgh	Delayed acrophase at baseline and greater annualized decline of Alzheimer disease-signature region cerebral glucose (AD-CM) are associated with cognitive dysfunction (p = .048). Significant positive association between the annualized decline of AD-CM and the

	compound-B positron emission tomography (PET), fluorodeoxyglucose-PET, magnetic resonance imaging, and MMSE.	reduction of Mini-Mental State Examination (MMSE) scores over a 2-year period (p = .04).
Actigrapyhically-defined sleep disturbance in Parkinson's disease is associated with differential aspects of cognitive functioning[32]	To identify the relationship between cognitive functioning and sleep disturbances n=95 with idiopathic PD and n=48 healthy controls were recruited, to measure nocturnal sleep efficacy they wore actigraphy for 2 weeks.	Sleep efficiency is associated with specific cognitive impairments. Working (r = .28) and verbal (r = .23) memory were significantly associated with sleep efficiency, but verbal fluency and attentional set-shifting were not associated.
**Other developments:**<br>- Smart homes with multi-room activity sensors show good correlation with clinician-scored cognitive status (r = .72)[33] and show moderate correlations with some in-lab tests and scales.[34]<br>- AUC of ≥0.93 for predicting MCI In multi-room environment over a period of 24 weeks.[35]<br>- In-house video monitoring can differentiate Alzheimer's disease from healthy controls[36]<br>- Computer use and more sleep were associated with better cognition.[37], whereas more time in kitchen and looking in fridge and cabinets is associated with worse cognition.[38]<br>- Higher latency in using punctuation and backspace keys was associated with worse processing speed between people with multiple sclerosis, but was not sensitive to progression over a 1-year period within participants.[39]<br>- Rest-activity rhythms associate with cognitive function during morning and afternoon activity.[40]		
**Anxiety**		
No supplementary studies.		
**Depressive symptoms**		
Mobile Phone Detection of Semantic Location and Its Relationship to Depression and Anxiety[41]	N = 208, using their mobile phone while their phone sensor data recorded for 6 weeks, to evaluate the relationship between semantic location visit patterns and depression and anxiety.	Low predictive value of semantic location (AUC 0.62). In combination with phone sensor data, AUC increased to 0.88.
Depressed Mood Prediction of Elderly People with a Wearable Band [42]	Proof-of-concept, n = 14 elderly without history of depression. 71 days of measurement using the *Empatica E4* wearable band, combining PPG and accelerometry, compared to depression scales.	Mean accuracy is 82.7% for PPG, 76.6% for accelerometry and 76.3% for the combination of PPG and accelerometry.
The relationship between mobile phone location sensor data and depressive symptom severity[43]	Validation study, n = 48 healthy students over a 10-week period. To investigate the correlation between GPS features/circadian activity and depressive symptoms. Revealing association at baseline, follow-up, and changes in symptoms severity over time.	GPS features can be a reliable predictor for severity of depressive symptoms. Measuring period of 10 weeks. Relatively short to be able to accurately predict onset of depression or depressive episodes. Severity of depressive symptoms is self-reported (PHQ-9).
A Sensor-Driven Visit Detection System in Older Adults Homes: Towards Digital Late-Life Depression Marker Extraction[44]	Longitudinal study, n = 13 healthy participants (age 86 +-7.23). Detection of home visits as a predictor for social isolation and late-life depression.	Small sample size, assumption that visits are well associated with common geriatric depression scale screening tool (ρ=-0.89, p=0.001)
**Other developments:**<br>- Objective (passive) sleep characteristics in PD are not predictive of depressive symptoms.[45]		

- Isolated HRV measurements has accuracy up to 83% in accuracy for depressive symptom prediction[46], although circadian rhythm in HRV was not associated with depressive symptoms.[47]<br>- Smartwatch-measured heart rate circadian rhythm is associated with depression severity.[48]<br>- Combination of passive smartphone use, voice samples, and social media use predicts depressive symptoms with a sensitivity of 0.75 and specificity of 0.79[49].<br>- More time at home is associated with depressive symptoms in young adults[50], and geographic location distribution associates with depressive symptoms in bipolar disorder.[51]<br>- No clear to low associations between sleep parameters, circadian rhythm and mood symptoms[48,52,53], although one recent study demonstrates predictive value of actigraphy-measured sleep and circadian rhythm disturbance on depressive symptoms in a longitudinal study.[54]		
**Olfactory dysfunction**		
No supplementary studies.		
**Hallucinations**		
No supplementary studies.		
**Color vision disturbances**		
No supplementary studies.		

Supplementary References

1. Carlson, C. *et al.* Bed-based instrumentation for unobtrusive sleep quality assessment in severely disabled autistic children. *Annu Int Conf IEEE Eng Med Biol Soc* **2016**, 4909-4912, doi:10.1109/embc.2016.7591828 (2016).
2. Li, S. & Chiu, C. A Smart Pillow for Health Sensing System Based on Temperature and Humidity Sensors. *Sensors* **18**, doi:http://dx.doi.org/10.3390/s18113664 (2018).
3. O'Dowd, S. T. *et al.* Longitudinal assessment of sleep in an incident Parkinson's disease cohort. *Movement Disorders* **31(Supplement 2)**, S117, doi:http://dx.doi.org/10.1002/mds.26688 (2016).
4. Breen, D. P. *et al.* Sleep and circadian rhythm regulation in early parkinson disease. *JAMA Neurology* **71(5)**, 589-595, doi:http://dx.doi.org/10.1001/jamaneurol.2014.65 (2014).
5. Moerman, C. *et al.* Towards a handy screening tool for REM sleep behaviour disorder: RDBAct algorithm from wrist actigraphy data. *Journal of Sleep Research. Conference: 25th Congress of the European Sleep Research Society, ESRS* **29**, doi:http://dx.doi.org/10.1111/jsr.13181 (2020).
6. Stefani, A. *et al.* Screening for idiopathic REM sleep behavior disorder: Usefulness of actigraphy. *Sleep* **41(6) (no pagination)**, doi:http://dx.doi.org/10.1093/sleep/zsy053 (2018).
7. Louter, M., Arends, J. B., Bloem, B. R. & Overeem, S. Actigraphy as a diagnostic aid for REM sleep behavior disorder in Parkinson's disease. *BMC Neurol* **14**, 76, doi:10.1186/1471-2377-14-76 (2014).
8. Naismith, S. L., Rogers, N. L., Mackenzie, J., Hickie, I. B. & Lewis, S. J. The relationship between actigraphically defined sleep disturbance and REM sleep behaviour disorder in Parkinson's Disease. *Clin Neurol Neurosurg* **112**, 420-423, doi:10.1016/j.clineuro.2010.02.011 (2010).
9. Memon, A. *et al.* Relationship between subjective and objective measures of sleepiness in Parkinson's disease. *Movement Disorders. Conference: 1st Pan American Parkinson's Disease and Movement Disorders Congress. Miami, FL United States* **32**, doi:http://dx.doi.org/10.1002/mds.26972 (2017).
10. Bolitho, S. J. *et al.* Objective measurement of daytime napping, cognitive dysfunction and subjective sleepiness in Parkinson's disease. *PLoS One* **8**, e81233, doi:10.1371/journal.pone.0081233 (2013).
11. Jeklin, A. T. *et al.* The association between heart rate variability, reaction time, and indicators of workplace fatigue in wildland firefighters. *International archives of occupational and environmental health* **94(5)**, 823-831, doi:http://dx.doi.org/10.1007/s00420-020-01641-3 (2021).
12. Tseng, V. W. S., Valliappan, N., Ramachandran, V., Choudhury, T. & Navalpakkam, V. Digital biomarker of mental fatigue. *npj Digital Medicine* **4(1) (no pagination)**, doi:http://dx.doi.org/10.1038/s41746-021-00415-6 (2021).
13. Sim, J. K., Yoon, S. & Cho, Y. H. Wearable Sweat Rate Sensors for Human Thermal Comfort Monitoring. *Scientific reports* **8(1)**, 1181, doi:http://dx.doi.org/10.1038/s41598-018-19239-8 (2018).
14. Santarelli, L. *et al.* Development of a Novel Wearable Ring-Shaped Biosensor. *Conference proceedings : ..* **Annual International Conference of the IEEE Engineering in Medicine and Biology Society. IEEE Engineering in Medicine and Biology Society. Annual Conference. 2018**, 3750-3753, doi:http://dx.doi.org/10.1109/EMBC.2018.8513330 (2018).
15. Nardelli, M., Vanello, N., Galperti, G., Greco, A. & Scilingo, E. P. Assessing the Quality of Heart Rate Variability Estimated from Wrist and Finger PPG: A Novel Approach Based on Cross-Mapping Method. *Sensors* **20**, doi:http://dx.doi.org/10.3390/s20113156 (2020).

16 Pandian, P. S. *et al.* Smart Vest: wearable multi-parameter remote physiological monitoring system. *Med Eng Phys* **30**, 466-477, doi:10.1016/j.medengphy.2007.05.014 (2008).
17 Reali, P., Tacchino, G., Rocco, G., Cerutti, S. & Bianchi, A. M. Heart Rate Variability from Wearables: A Comparative Analysis Among Standard ECG, a Smart Shirt and a Wristband. *Studies in health technology and informatics* **261**, 128-133 (2019).
18 Sarhaddi, F. *et al.* A comprehensive accuracy assessment of Samsung smartwatch heart rate and heart rate variability. *PLoS One* **17**, e0268361, doi:10.1371/journal.pone.0268361 (2022).
19 Schultz, R. E. Smartphone App for In-home Uroflowmetry. *Urol Pract* **9**, 524-530, doi:10.1097/UPJ.0000000000000338 (2022).
20 Edgar, R., Trip, E. J., Wolterink, G. J. W., Veltink, P. H. & Beck, J. J. H. New methods for the monitoring of nocturnal erections. *Int J Impot Res*, doi:10.1038/s41443-020-00365-9 (2020).
21 Aqajari, S. A. H. *et al.* Pain Assessment Tool With Electrodermal Activity for Postoperative Patients: Method Validation Study. *JMIR mHealth and uHealth* **9(5)**, e25258, doi:http://dx.doi.org/10.2196/25258 (2021).
22 Ummel, J. D. *et al.* Kick Ring LL: A Multi-Sensor Ring Capturing Respiration, Electrocardiogram, Oxygen Saturation, and Skin Temperature(1). *Annu Int Conf IEEE Eng Med Biol Soc* **2020**, 4394-4397, doi:10.1109/embc44109.2020.9176654 (2020).
23 Ibrahim, B. & Jafari, R. Cuffless Blood Pressure Monitoring from an Array of Wrist Bio-Impedance Sensors Using Subject-Specific Regression Models: Proof of Concept. *IEEE Trans Biomed Circuits Syst* **13**, 1723-1735, doi:10.1109/tbcas.2019.2946661 (2019).
24 Gaurav, A., Maheedhar, M., Tiwari, V. N. & Narayanan, R. Cuff-less PPG based continuous blood pressure monitoring: a smartphone based approach. *Conference proceedings : ..* **Annual International Conference of the IEEE Engineering in Medicine and Biology Society. IEEE Engineering in Medicine and Biology Society. Annual Conference. 2016**, 607-610, doi:http://dx.doi.org/10.1109/EMBC.2016.7590775 (2016).
25 Rachim, V. P. & Chung, W. Y. Multimodal Wrist Biosensor for Wearable Cuff-less Blood Pressure Monitoring System. *Sci Rep* **9**, 7947, doi:10.1038/s41598-019-44348-3 (2019).
26 Park, S. M. *et al.* A mountable toilet system for personalized health monitoring via the analysis of excreta. *Nature Biomedical Engineering* **4(6)**, 624-635, doi:http://dx.doi.org/10.1038/s41551-020-0534-9 (2020).
27 Maqbool, S., Parkman, H. P. & Friedenberg, F. K. Wireless capsule motility: comparison of the SmartPill GI monitoring system with scintigraphy for measuring whole gut transit. *Dig Dis Sci* **54**, 2167-2174, doi:10.1007/s10620-009-0899-9 (2009).
28 Pehlivan, M. *et al.* An electronic device measuring the frequency of spontaneous swallowing: digital phagometer. *Dysphagia* **11**, 259-264, doi:10.1007/bf00265212 (1996).
29 Hadley, A. J., Krival, K. R., Ridgel, A. L., Hahn, E. C. & Tyler, D. J. Neural network pattern recognition of lingual-palatal pressure for automated detection of swallow. *Dysphagia* **30**, 176-187, doi:10.1007/s00455-014-9593-y (2015).
30 Akl, A. *et al.* Clustering Home Activity Distributions for Automatic Detection of Mild Cognitive Impairment in Older Adults. *J Ambient Intell Smart Environ* **8**, 437-451, doi:10.3233/AIS-160385 (2016).
31 Jeon, S. Y. *et al.* Circadian rest-activity rhythm and longitudinal brain changes underlying late-life cognitive decline. *Psychiatry Clin Neurosci* **77**, 205-212, doi:10.1111/pcn.13521 (2023).

32 Gunn, D. G., Naismith, S. L., Bolitho, S. J. & Lewis, S. J. Actigraphically-defined sleep disturbance in Parkinson's disease is associated with differential aspects of cognitive functioning. *J Clin Neurosci* **21**, 1112-1115, doi:10.1016/j.jocn.2013.09.017 (2014).

33 Dawadi, P. N., Cook, D. J. & Schmitter-Edgecombe, M. Automated Cognitive Health Assessment From Smart Home-Based Behavior Data. *IEEE J Biomed Health Inform* **20**, 1188-1194, doi:10.1109/JBHI.2015.2445754 (2016).

34 Alberdi, A. *et al.* Smart Home-Based Prediction of Multidomain Symptoms Related to Alzheimer's Disease. *IEEE J Biomed Health Inform* **22**, 1720-1731, doi:10.1109/JBHI.2018.2798062 (2018).

35 Akl, A., Taati, B. & Mihailidis, A. Autonomous unobtrusive detection of mild cognitive impairment in older adults. *IEEE Trans Biomed Eng* **62**, 1383-1394, doi:10.1109/TBME.2015.2389149 (2015).

36 Sacco, G. *et al.* Detection of activities of daily living impairment in Alzheimer's disease and mild cognitive impairment using information and communication technology. *Clinical Interventions in Aging* **7**, 539-549, doi:http://dx.doi.org/10.2147/CIA.S36297 (2012).

37 Bernstein, J. P. K. *et al.* Unobtrusive, in-home assessment of older adults' everyday activities and health events: associations with cognitive performance over a brief observation period. *Neuropsychology, development, and cognition* **Section B, Aging, neuropsychology and cognition.**, 1-18, doi:http://dx.doi.org/10.1080/13825585.2021.1917503 (2021).

38 Lussier, M. *et al.* Smart Home Technology: A New Approach for Performance Measurements of Activities of Daily Living and Prediction of Mild Cognitive Impairment in Older Adults. *J Alzheimers Dis* **68**, 85-96, doi:10.3233/JAD-180652 (2019).

39 Lam, K. H. *et al.* The Use of Smartphone Keystroke Dynamics to Passively Monitor Upper Limb and Cognitive Function in Multiple Sclerosis: Longitudinal Analysis. *J Med Internet Res* **24**, e37614, doi:10.2196/37614 (2022).

40 Antonsdottir, I. M. *et al.* 24 h Rest/Activity Rhythms in Older Adults with Memory Impairment: Associations with Cognitive Performance and Depressive Symptomatology. *Adv Biol (Weinh)*, e2300138, doi:10.1002/adbi.202300138 (2023).

41 Saeb, S., Lattie, E. G., Kording, K. P. & Mohr, D. C. Mobile Phone Detection of Semantic Location and Its Relationship to Depression and Anxiety. *JMIR Mhealth Uhealth* **5**, e112, doi:10.2196/mhealth.7297 (2017).

42 Choi, J., Lee, S., Kim, S., Kim, D. & Kim, H. Depressed Mood Prediction of Elderly People with a Wearable Band. *Sensors (Basel)* **22**, doi:10.3390/s22114174 (2022).

43 Saeb, S., Lattie, E. G., Schueller, S. M., Kording, K. P. & Mohr, D. C. The relationship between mobile phone location sensor data and depressive symptom severity. *PeerJ* **2016(9) (no pagination)**, doi:http://dx.doi.org/10.7717/peerj.2537 (2016).

44 Schutz, N. *et al.* A Sensor-Driven Visit Detection System in Older Adults Homes: Towards Digital Late-Life Depression Marker Extraction. *IEEE Journal of Biomedical and Health Informatics.*, doi:http://dx.doi.org/10.1109/JBHI.2021.3114595 (2021).

45 Mulryan, P., Affonso, S. & Sullivan, A. Sleep and depression in Parkinson's disease: Investigating the relationship between sleep and depression using a combination of subjective and objective sleep assessment methods. *Annals of Neurology* **88(SUPPL 25)**, S196, doi:http://dx.doi.org/10.1002/ana.25865 (2020).

46 Coutts, L. V., Plans, D., Brown, A. W. & Collomosse, J. Deep learning with wearable based heart rate variability for prediction of mental and general health. *Journal of Biomedical Informatics* **112 (no pagination)**, doi:http://dx.doi.org/10.1016/j.jbi.2020.103610 (2020).

47 Lee, D. *et al.* Changes in the Circadian Rhythm of High-Frequency Heart Rate Variability Associated With Depression. *J Korean Med Sci* **38**, e142, doi:10.3346/jkms.2023.38.e142 (2023).

48 Siddi, S. *et al.* The usability of daytime and night-time heart rate dynamics as digital biomarkers of depression severity. *Psychol Med* **53**, 3249-3260, doi:10.1017/S0033291723001034 (2023).

49 Dogrucu, A. *et al.* Moodable: On feasibility of instantaneous depression assessment using machine learning on voice samples with retrospectively harvested smartphone and social media data. *Smart Health* **17 (no pagination)**, doi:http://dx.doi.org/10.1016/j.smhl.2020.100118 (2020).

50 Chow, P. I. *et al.* Using Mobile Sensing to Test Clinical Models of Depression, Social Anxiety, State Affect, and Social Isolation Among College Students. *Journal of medical Internet research* **19(3)**, e62, doi:http://dx.doi.org/10.2196/jmir.6820 (2017).

51 Palmius, N. *et al.* Detecting bipolar depression from geographic location data. *IEEE Transactions on Biomedical Engineering* **64(8)**, 1761-1771, doi:http://dx.doi.org/10.1109/TBME.2016.2611862 (2017).

52 Braund, T. A. *et al.* Smartphone Sensor Data for Identifying and Monitoring Symptoms of Mood Disorders: A Longitudinal Observational Study. *JMIR Ment Health* **9**, e35549, doi:10.2196/35549 (2022).

53 Hayashi, M., Takeshima, M., Hosoya, T. & Kume, Y. 24-Hour Rest-Activity Rhythm in Middle-Aged and Older Persons with Depression. *Int J Environ Res Public Health* **20**, doi:10.3390/ijerph20075275 (2023).

54 de Feijter, M., Kocevska, D., Ikram, M. A. & Luik, A. I. The bidirectional association of 24-h activity rhythms and sleep with depressive symptoms in middle-aged and elderly persons. *Psychol Med* **53**, 1418-1425, doi:10.1017/S003329172100297X (2023).
